# Biotin-tagged proteins: Reagents for efficient ELISA-based serodiagnosis and phage display-based affinity selection

**DOI:** 10.1371/journal.pone.0191315

**Published:** 2018-01-23

**Authors:** Vaishali Verma, Charanpreet Kaur, Payal Grover, Amita Gupta, Vijay K. Chaudhary

**Affiliations:** 1 Centre for Innovation in Infectious Disease Research, Education and Training (CIIDRET), University of Delhi South Campus, New Delhi, India; 2 Department of Biochemistry, University of Delhi South Campus, New Delhi, India; USDA-ARS, UNITED STATES

## Abstract

The high-affinity interaction between biotin and streptavidin has opened avenues for using recombinant proteins with site-specific biotinylation to achieve efficient and directional immobilization. The site-specific biotinylation of proteins carrying a 15 amino acid long Biotin Acceptor Peptide tag (BAP; also known as AviTag) is effected on a specific lysine either by co-expressing the *E*. *coli* BirA enzyme *in vivo* or by using purified recombinant *E*. *coli* BirA enzyme in the presence of ATP and biotin *in vitro*. In this paper, we have designed a T7 promoter-lac operator-based expression vector for rapid and efficient cloning, and high-level cytosolic expression of proteins carrying a C-terminal BAP tag in *E*. *coli* with TEV protease cleavable N-terminal deca-histidine tag, useful for initial purification. Furthermore, a robust three-step purification pipeline integrated with well-optimized protocols for TEV protease-based H10 tag removal, and recombinant BirA enzyme-based site-specific *in vitro* biotinylation is described to obtain highly pure biotinylated proteins. Most importantly, the paper demonstrates superior sensitivities in indirect ELISA with directional and efficient immobilization of biotin-tagged proteins on streptavidin-coated surfaces in comparison to passive immobilization. The use of biotin-tagged proteins through specific immobilization also allows more efficient selection of binders from a phage-displayed naïve antibody library. In addition, for both these applications, specific immobilization requires much less amount of protein as compared to passive immobilization and can be easily multiplexed. The simplified strategy described here for the production of highly pure biotin-tagged proteins will find use in numerous applications, including those, which may require immobilization of multiple proteins simultaneously on a solid surface.

## Introduction

Over the last two decades, specific and efficient capture of biotin-tagged proteins on streptavidin surface by virtue of extremely high-affinity interaction has found use in a diverse range of biological applications [1, 2] including immunoassays [3–6], phage display-based affinity selection [7, 8], and affinity-based purification [9, 10]. Conventional approaches for immobilization of proteins involve their passive adsorption on polystyrene surfaces. Besides being non-specific, it also leads to denaturation of proteins, which is often undesirable [11, 12]. Particularly, during phage display-based affinity selection procedures, passive immobilization may significantly alter the structure of the bait proteins, thereby yielding binders that may not recognize the native protein [13].

Furthermore, passive immobilization of proteins may be highly variable depending on the composition and exposure of hydrophobic patches of proteins [11]. This phenomenon may have more pronounced effect during immobilization of multiple proteins as a mixture, as is the case in the detection of infectious diseases. For example, according to the literature, diagnosis of tuberculosis requires testing with a combination of proteins in ELISA or lateral flow-based antibody detection tests [14–17]. The differential coating characteristics during passive immobilization of proteins in a mixture would affect the assay sensitivity.

A method that allows efficient immobilization of various proteins in a mixture, irrespective of their differential properties can address this issue. One such method is the use of streptavidin-coated surface for uniform and efficient immobilization of biotin-tagged proteins, while preserving their conformation. The pre-requisite to exploit this specific immobilization strategy would be the production of biotin-tagged proteins. Several methods have been described for covalent attachment of biotin to the proteins (a process referred as “biotinylation”). One method to achieve this is chemical-based biotinylation, which employs reagents that target amine, sulphydryl or carboxyl functional groups present in proteins [1]. However, this process lacks reproducibility and varies from protein to protein, based on the availability of the functional groups. It can also lead to the loss of protein activity or disruption of protein structure [13, 18].

These concerns can be addressed using methods to achieve site-specific enzymatic biotinylation of proteins under *in vivo* or *in vitro* conditions [18]. Bacterium *Escherichia coli (E*. *coli)* naturally produces a protein known as Biotin Carboxyl Carrier Protein (BCCP), which is a subunit of acetyl-CoA carboxylase enzyme. BCCP is specifically biotinylated by *E*. *coli* biotin holoenzyme synthetase (BirA), which catalyzes covalent linkage of biotin to an epsilon amine group of a specific lysine residue in BCCP in the presence of ATP [19–22]. This knowledge was used to decipher 70–80 amino acid domain of BCCP that serves as the target of BirA [21, 22]. However, the attachment of such a large sequence may affect the properties of the recombinant fusion protein, and the biotinylation site may not be always accessible leading to reduction in the biotinylation efficiency. Schatz and co-workers identified a 14-mer peptide mimic that contains a lysine residue, which is the target for site-specific biotinylation by BirA enzyme and is functionally similar to its natural substrate BCCP [23]. This was later extended to a 15-mer peptide sequence “GLNDIFEAQ**K**IEWHE” (Biotin Acceptor Peptide; BAP; also known as AviTag), which can be translationally fused at either N or C-terminus of proteins to produce biotin-tagged proteins [18]. Typically, *in vivo* biotinylation methods involve co-expression of BAP-tagged proteins and BirA enzyme using appropriate vector systems to obtain biotin-tagged proteins [18]. However, this approach may require optimization of expression levels of the protein and BirA enzyme, and the extent of biotinylation may vary if the target protein is insoluble and forms inclusion bodies or is targeted for expression in *E*. *coli* periplasm (as BirA is cytosolic enzyme). Under such scenarios, the *in vitro* biotinylation reaction can be performed using purified BAP-tagged protein with recombinant BirA enzyme in the presence of biotin and ATP under appropriate reaction conditions, followed by the purification of the biotinylated protein [18, 24].

In this paper, using five secretory proteins of *Mycobacterium tuberculosis (M*. *tuberculosis)* H37Rv, namely, MTC28 (Rv0040c), MPT63 (Rv1926c), MPT64 (Rv1980c), Ag85A (Rv3804c), and Ag85B (Rv1886c), we have described a streamlined workflow for cloning, cytosolic expression, and purification of the expressed protein, followed by its *in vitro* biotinylation to obtain highly pure recombinant protein carrying biotin attached to the BAP tag present at the C-terminus. A T7 promoter-lac operator-based IPTG/lactose inducible vector system employing rapid and high-throughput restriction enzyme-free cloning of genes has been developed for auto-induction-based cytosolic expression of recombinant proteins carrying N-terminal deca-histidine tag (H10), TEV protease site, and C-terminal BAP tag with appropriate spacers between different elements. Furthermore, a robust three-step chromatography pipeline integrated with well-optimized and efficient protocols for TEV protease-based H10 tag removal, and recombinant *E*. *coli* BirA enzyme-based site-specific *in vitro* biotinylation has been described to obtain purified proteins (devoid of the N-terminal H10 tag) carrying biotin residue at the C-terminus. Most importantly, the utility of these biotin-tagged recombinant proteins has been exemplified by comparison of passive versus specific protein immobilization in the context of indirect ELISA, and phage display-based affinity selection.

## Materials

*Escherichia coli* strains BL21 (DE3) RIL (B F^−^*ompT hsdS* (r_B_^−^m_B_^−^) *dcm*+ Tet^r^ *gal l* (DE3) *endA* Hte *[argU ileY leuW* Cam^r^), and TOP10F’ (F’ [*lacI*^*q*^ Tn10 (*tet*^R^)] *mcr*A Δ(*mrr-hsd*RMS-*mcr*BC) φ80*lac*ZΔM15 Δ*lac*X74 *deo*R *nup*G *rec*A1 *ara*D139 Δ(*ara-leu*)7697 *gal*U *gal*K *rps*L(*Str*^R^) *end*A1 λ^-^) were obtained from commercial sources. ATP (disodium salt) was obtained from Roche, Mannheim, Germany. HRP-conjugated Goat anti-Mouse IgG (H+L) antibody and HRP-conjugated Goat anti-Rabbit IgG (H+L) antibody were obtained from Jackson ImmunoResearch Laboratories Inc, PA, US. Nunc-polystyrene Immunotubes (cat. no. 470319), Nunc Maxisorp polystyrene 384 well plates with clear, flat bottom (cat. no. 464718), Nunc Immobilizer streptavidin 384 well plates with clear, flat bottom, and covalently coated streptavidin (cat no. 436017), MyOne streptavidin T1 and M-280 streptavidin Dynabeads were from Thermo Fisher Scientific, Waltham, US. D-Biotin and all other standard chemicals were obtained from Affymetrix, CA, USA. Oligonucleotides were obtained from Sigma-Aldrich, Bangalore, India. Restriction enzymes, T4 DNA ligase, and T4 DNA polymerase were obtained from NEB, Ipswich, MA, USA. PfuUltra II Fusion HS DNA polymerase was obtained from Agilent Technologies, Santa Clara, US. Chromatography resins and columns were obtained from GE Healthcare Life Sciences, Uppsala, Sweden. Reagents for polyacrylamide gel electrophoresis were obtained from Bio-Rad, Hercules, USA.

A phage-displayed naive human antibody library comprising of 10 billion clones in scFv (single chain fragment variable) format was available in the laboratory. This library was stored as eight AGM13 helper phage [25] rescued, and PEG-purified phage sub-libraries, 4 each with kappa and lambda light chains (each phage sub-library represents ~ 1.25 x 10^9^ clones). Recombinant hexa-histidine-tagged TEV protease (H6-TEV protease) carrying mutation S219V for reduced autolysis activity and improved catalytic activity was expressed from vector pRK793 (obtained from Addgene; plasmid 8827), and purified in-house following protocols described by Tropea et al. [26]. Recombinant N-terminal deca-histidine-tagged *E*. *coli* BirA enzyme (H10-BirA) was produced in-house using a T7 promoter-lac operator-based expression vector, pVLExpBirA4231, carrying sequence encoding *E*. *coli* BirA enzyme, and subjecting its transformants in *E*. *coli* BL21 (DE3) RIL to auto-induction in ZYM5052 medium at 18°C for high-level cytosolic expression. The H10-BirA protein was purified from cytosolic fraction using a two-step protocol involving affinity chromatography on Ni Sepharose Fast Flow resin (NiFF), and gel-filtration chromatography on Superdex 200 to obtain tens of milligram of pure ~ 38 kDa monomeric protein. Bacterially expressed and purified N-terminal deca-histidine-tagged recombinant mycobacterial protein H10-MTC28 (Rv0040c) was available in-house at concentration 2 mg/ml in 20 mM phosphate buffer, pH 7.5. Purified mouse monoclonal antibodies MTC28-13 (MTC28 specific), MPT63-03 (MPT63 specific), MPT64-33 (MPT64 specific), Ag85-14 (Ag85A specific), Ag85-11 (Ag85B specific), and Ag85-12 (Ag85A and Ag85B specific) derived from hybridoma clones were available in-house. Rabbit polyclonal sera against purified polyhistidine-tagged proteins (without BAP tag) were produced by a commercial source, Bangalore Genei, India (Merck), and purified in the laboratory.

## Methods

### Construction of expression vector pVMExp14367

Vector pVMExp14367 is a medium copy number T7 promoter-lac operator-based expression vector containing sequence encoding N-terminal deca-histidine tag (H10), Tobacco Etch Virus (TEV) protease cleavage site, 2.0 Kb SacR-SacB gene cassette encoding levansucrase protein of *Bacillus subtilis* flanked by two appropriately oriented BsaI sites, and 15 amino acid C-terminal Biotin Acceptor Peptide tag (BAP) (Genbank accession number MG599491). In between the functional tags/protease site, there are glycine-serine rich spacer sequences of appropriate lengths. The vector backbone comprises of the ColE1 origin of replication (ori) with deletion of *rop* gene, filamentous phage origin of replication (f ori), beta-lactamase gene as a selection marker and lac repressor (*lacI*). This vector design is compatible with highly efficient restriction enzyme-free cloning of the genes as described before [27]. The vector pVMExp14367 was constructed by assembly of several components encoding T7 promoter-lac operator, H10 tag, TEV protease cleavage site, ampicillin resistance marker; beta-lactamase gene, and ColE1 ori from a set of intermediate vectors, and synthetic DNA sequences available in the laboratory. SacR-SacB gene cassette was obtained as a synthetic gene from Geneart (Thermo Fisher Scientific, Waltham, US), and BAP tag was assembled using duplex of oligonucleotides encoding 15 amino acid sequence. The final recombinant was sequenced using ABI 3730 XL DNA sequencing platform (Applied Biosystems, Thermo Fisher Scientific, Waltham, USA).

### Cloning of five *M*. *tuberculosis* genes in the expression vector pVMExp14367

DNA encoding five *M*. *tuberculosis* H37Rv proteins, namely, MTC28 (Rv0040c), MPT63 (Rv1926c), MPT64 (Rv1980c), Ag85A (Rv3804c), and Ag85B (Rv1886c) was amplified from respective templates (S1A Fig) available in the laboratory using 5’ and 3’ gene-specific primers carrying 7 base extensions to append sequences required for restriction enzyme-free cloning [27]. For cloning, the preparation of inserts was performed using “column method” as described before [27]. The BsaI-digested linearized vector was also prepared as described before [27]. The ligation reaction was set up in 10 μl reaction volume containing 1.0 μl of the T4 DNA polymerase-treated insert (~ 50 ng), 50 ng BsaI-digested linearized and purified pVMExp14367 vector, and 200 U of T4 DNA Ligase (400 U/μl) in 1 x ligation buffer. The ligation reaction was carried out at 16°C for 1 hr and 37°C for 1 hr followed by inactivation of T4 DNA ligase at 65°C for 10 min. The ligation reaction (1 μl) was diluted to 10 μl in distilled water, and 500 pg equivalent ligated vector was electroporated in 25 μl electrocompetent *E*. *coli* BL21 (DE3) RIL cells (electroporation efficiency ~ 5 x 10^8^/μg pGEM DNA), and plated on MDAG plates containing 100 μg/ml ampicillin and 30 μg/ml chloramphenicol (MDAGAmp_100_Cm_30_). Three transformants of each construct were checked by sequencing on ABI 3730 XL sequencing platform (Applied Biosystems, Thermo Fisher Scientific, Waltham, USA), and clones with correct sequence were chosen for expression studies.

### Expression and localization of the proteins

Small-scale expression and localization was carried out to determine the yield of soluble protein. For this, protein expression was performed by auto-induction in 50 ml ZYM5052 media [28]. The clones from MDAGAmp_100_Cm_30_ plates were inoculated in 3 ml MDAGAmp_100_Cm_30_ liquid media (primary culture) and were grown at 30°C, 250 rpm for 18 hr. The primary culture was diluted 100 fold in 50 ml ZYM5052 media containing 100 μg/ml ampicillin and 30 μg/ml chloramphenicol (ZYM5052Amp_100_Cm_30_) and grown with shaking at 250 rpm at 30°C for 2 hr, 24°C for 4 hr, and 18°C for 16 hr. The cells were harvested and re-suspended in 25 ml of 1 x TLB (50 mM Tris-HCl, pH 7.5 buffer containing 500 mM NaCl) supplemented with lysozyme at a final concentration of 200 μg/ml and PMSF at a final concentration of 0.1 mM, and incubated on ice for 30 min. Cells were lysed using sonication (Misonix, Model XL 3000). The lysate was centrifuged at 22,000*g* for 30 min at 4°C to obtain the supernatant (HSS; High Speed Supernatant), which was further centrifuged at 50,000*g* for 2 hr at 4°C, and the resulting supernatant containing soluble proteins was named as HHSS (High-High Speed Supernatant). For localization of the recombinant proteins, different sub-cellular fractions were analyzed on 0.1% SDS—8–20% polyacrylamide gradient gel under reducing conditions.

Based on the yield of soluble recombinant proteins in HHSS as determined by Coomassie brilliant blue R-250 dye stained gel, an appropriate volume of preparative-scale culture was set up for all the five proteins under the same expression conditions as described above. After auto-induction, the cells were harvested and resuspended in half the volume of 1 x TLB (without the addition of lysozyme and PMSF), followed by homogenization of cells using PANDA high-pressure homogenizer (GEA Niro Soavi) at 800 bars for 6 cycles as per the manufacturer’s instructions. The lysate was centrifuged, and HHSS was prepared as described for the small-scale expression. The HHSS fraction containing soluble proteins was then subjected to three-step chromatography protocol to obtain purified protein preparations.

### Purification of the proteins

The entire purification procedure was performed at 4–8°C using appropriate chromatography columns attached to the AKTA Explorer 100 system (GE Healthcare Life Sciences, Uppsala, Sweden). During chromatography, the eluted protein was monitored using absorbance at 280 nm; fractions (of appropriate volumes depending on the type of chromatography) were collected and analyzed using SDS-PAGE under reducing conditions. Based on the purity and yield of the proteins, fractions were pooled.

For the purification of H10-T-MTC28-BAP protein, 280 ml HHSS containing approximately 330 mg of recombinant protein in 1 x TLB with 20 mM imidazole was filtered through 0.45 μ membrane, and applied on 20 ml Ni Sepharose Fast Flow (NiFF) resin packed in HR16/10 column (pre-equilibrated with 1 x TLB containing 20 mM imidazole) at a flow rate of 3 ml/min. After loading of the sample, the column was washed with 60 ml (3 CV) of 1 x TLB containing 20 mM imidazole, followed by 160 ml (8 CV) of 1 x TLB containing 50 mM imidazole at a flow rate of 3.5 ml/min. Finally, the bound protein was eluted with 1 x TLB containing 300 mM imidazole at 2 ml/min and 2 ml fractions were collected. The fractions were analyzed using 0.1% SDS-12.5% PAGE and those containing desired protein were pooled (NiFF pool).

The NiFF pool was further purified by gel-filtration chromatography on 480 ml Superdex 75 column pre-equilibrated with 20 mM Tris-HCl, pH 8.0 containing 50 mM NaCl. The NiFF pool was loaded at a flow rate of 3 ml/min, the column was subsequently developed at a flow rate of 3 ml/min, and 4 ml fractions were collected. The fractions were analyzed using 0.1% SDS-12.5% PAGE and those containing relatively pure protein were pooled (GFC pool).

Next, to obtain the protein devoid of N-terminal H10 tag (i.e., MTC28-BAP from H10-T-MTC28-BAP), the purified protein in the GFC pool was subjected to treatment with H6-TEV protease. For this, approximately 200 mg of H10-T-MTC28-BAP protein was subjected to digestion with 2 mg H6-TEV protease (~ 1:100 ratio of TEV protease:substrate; w/w) in a reaction volume of 45 ml in 1 x TEV reaction buffer (50 mM Tris-HCl, pH 8.0, 0.5 mM EDTA, 1 mM DTT) for 3 hr at 30°C. After completion of the reaction, sample equivalent to ~ 8 μg protein was analyzed on 0.1% SDS-12.5% PAGE to determine the extent of cleavage. After completion of the digestion process, 5 ml of 10 x loading supplement (2.5 M NaCl, 10 mM MgCl_2_, 50 mM Tris-HCl pH 8.0, 200 mM imidazole) was added to the reaction. The resultant digestion mixture containing 20 mM imidazole in 1 x TLB, was applied on a 20 ml NiFF resin packed in HR16/10 column (pre-equilibrated with 1 x TLB containing 20 mM imidazole) at a flow rate of 1 ml/min. Flow-through fractions containing MTC28-BAP protein were pooled as NiFF pool after TEV protease Treatment (NiFF-TT pool).

Finally, the NiFF-TT pool containing MTC28-BAP protein was subjected to anion-exchange chromatography. For this, the NiFF-TT pool was desalted using Sephadex G25 (140 ml resin packed in XK 26/40 column), pre-equilibrated with 20 mM Tris-HCl, pH 8.0 (buffer A). For desalting the entire NiFF-TT pool (~ 60 ml), the same column was used twice (30 ml sample was desalted per run) with column re-equilibration in between the two runs. For anion-exchange chromatography, the desalted NiFF-TT pool (~ 80 ml) was applied on 20 ml Q Sepharose High Performance (QHP) resin (packed in HR16/10 column; pre-equilibrated with buffer A) at a flow rate of 4 ml/min. The QHP column was washed with 60 ml (3 CV) of buffer A, elution was performed with 100 ml (5 CV) linear gradient of 0–0.5 M NaCl in buffer A at 4 ml/min, and 2 ml fractions were collected. Fractions were analyzed using 0.1% SDS-12.5% PAGE and fractions containing pure protein were pooled (named as QHP pool). The protein concentration of QHP pool was estimated by measuring absorbance at 280 nm. The remaining four proteins were also purified employing the same process as described above for MTC28-BAP protein.

### *In vitro* biotinylation of purified proteins using *E*. *coli* H10-BirA enzyme

For biotinylation of the proteins, the QHP pool containing purified C-terminal BAP-tagged protein was subjected to *in vitro* biotinylation using recombinant deca-histidine-tagged BirA enzyme (H10-BirA). For each protein, 10 mg of the QHP purified pool (~ 2 ml) was subjected to buffer-exchange using 13 ml Sephadex G25 (fine) syringe column equilibrated in 50 mM Tris-HCl, pH 8.0 buffer. The protein eluted in approximately 4.5–5 ml volume. The *in vitro* biotinylation reaction was set up in approximately 6 ml volume containing 10 mg of purified C-terminal BAP-tagged protein (substrate), with 10 mM ATP, 10 mM MgCl_2_, 50 μM D-biotin, and 0.2 mg of recombinant H10-BirA enzyme (to achieve enzyme to substrate ratio of 1:50, w/w), and incubated at 30°C for 3 hr. After completion, 660 μl of 10 x loading supplement (2.5 M NaCl, 10 mM MgCl_2_, 50 mM Tris-HCl pH 8.0, 200 mM imidazole) was added to the reaction and applied on 1.2 ml NiFF column at a flow rate of 0.2 ml/min and 0.5 ml flow-through fractions were collected. This allowed binding of H10-BirA enzyme to the column, and the biotinylated protein collected in the flow-through fractions. The flow-through fractions were pooled and final volume was reduced to approximately 2 ml using amicon-ultra-4 centrifugal filter unit (10 kDa Cut Off; Merck Millipore) followed by desalting on 13 ml Sephadex G25 (fine) syringe column in 1 x PBS buffer (20 mM phosphate, pH 7.5 containing 150 mM NaCl) to obtain purified biotinylated proteins. The protein concentration was estimated by measuring absorbance at 280 nm. Purified biotinylated proteins were analyzed using 0.1% SDS-12.5% PAGE under reducing conditions.

### Determination of the extent of biotinylation in biotin-tagged proteins

To determine the extent of biotinylation, 25 μg of each biotin-tagged protein was adsorbed on 50 μl Streptavidin Sepharose HP beads (taken in 3–5 fold excess based on the binding capacity to ensure complete adsorption) by constant mixing for 1 hr at 25°C in a 2 ml tube (click cap, clear, round bottom microtubes, Treff Lab, Switzerland). The supernatant (labeled as ‘after’ adsorption fraction) was collected after centrifugation of mixture at 12,000*g* for 5 min at 25°C. Separately, a similarly diluted sample of each biotin-tagged protein was prepared and labeled as ‘before’ adsorption fraction. To determine the amount of protein remaining after adsorption, both ‘before’ and ‘after’ adsorption fractions were tested using indirect ELISA with protein-specific monoclonal antibodies to determine the amount of protein. For this, 384 well Nunc Immobilizer streptavidin-coated plate was washed thrice with PBST (1 x PBS with 0.05% Tween-20), and coated with 25 μl each of 7 point 3-fold dilutions of ‘before’ (range ~ 1:100–1:100K) and ‘after’ (range ~ 1:10–1:10K) adsorption fractions (prepared in 1 x PBS) for 2 hr at 25°C. Wells were washed thrice with PBST and blocked with 2% BSA-PBST for 1 hr at 25°C. After blocking, plate was washed thrice with PBST and proteins were probed with 100 ng/ml of respective protein-specific monoclonal antibodies (MAb MTC28-13 for MTC28, MAb MPT63-03 for MPT63, MAb MPT64-33 for MPT64, and MAb Ag85-12 for Ag85A and Ag85B) diluted in 0.1% BSA-PBST for 1 hr at 25°C. The plate was washed thrice with PBST, and HRP-conjugated Goat anti-Mouse IgG (H+L) antibody diluted 1:5000 times in 0.1% BSA-PBST was added for 1 hr at 25°C. Finally, after three washes each with PBST and 1 x PBS, the reaction was revealed by 25 μl TMB substrate (Seramun Diagnostics, Berlin, Germany). Following, incubation in the dark for 15 min at 25°C, the reaction was terminated by addition of 25 μl 1 N H_2_SO_4_ and absorbance was measured at 450 nm using ELISA plate reader (SpectraMax M5; Molecular Devices, Sunnyvale, CA, USA). The extent of biotinylation was calculated based on the fold reduction in the reactivity after adsorption on streptavidin beads.

### Indirect ELISA with biotin-tagged antigens using mouse monoclonal or rabbit polyclonal antibodies

To compare the coating efficiency of biotinylated proteins individually, different dilutions of biotin-tagged proteins (7 point 3-fold dilutions; range ~ 1 μg/ml to ~ 1.3 ng/ml) were prepared in 1 x PBS, and 25 μl of each was added to either 384 well Nunc Immobilizer streptavidin-coated plate (pre-washed thrice with PBST) for 2 hr at 25°C, or 384 well Nunc Maxisorp polystyrene plate for 2 hr at 37°C. Wells were washed thrice with PBST and blocked with 2% BSA-PBST for 1 hr at 25°C. After blocking, the plates were washed thrice with PBST and the proteins were probed either with 100 ng/ml of protein-specific purified mouse monoclonal antibodies (MAb MTC28-13 for MTC28, MAb MPT63-03 for MPT63, MAb MPT64-33 for MPT64, MAb Ag85-14 for Ag85A, and MAb Ag85-11 for Ag85B), or with 100 ng/ml of protein-specific purified rabbit polyclonal antibodies (both diluted in 0.1% BSA-PBST) for 1 hr at 25°C. Following this, the plates were washed thrice with PBST, and HRP-conjugated Goat anti-Mouse IgG (H+L) (diluted 1:5000 times in 0.1% BSA-PBST), or HRP-conjugated Goat anti-Rabbit IgG (H+L) antibodies (diluted 1:10,000 times in 0.1% BSA-PBST) were added for 1 hr at 25°C to probe the bound mouse monoclonal or rabbit polyclonal antibodies, respectively. Remaining steps were performed as described above.

To compare the coating efficiency of a mixture of biotin-tagged proteins, 10 μg each of 5 proteins (MTC28-Bio, MPT63-Bio, MPT64-Bio, Ag85A-Bio and Ag85B-Bio) was mixed (i.e., equal ratio, w/w), and 7 point 3-fold dilutions of resultant mixture (range ~ 5 μg/ml to ~ 7 ng/ml of the mixture, equivalent to ~ 1 μg/ml to ~ 1.3 ng/ml of individual protein) were captured on 384 well Nunc Immobilizer streptavidin-coated plate (pre-washed thrice with PBST) for 2 hr at 25°C, or 384 well Nunc Maxisorp polystyrene plate for 2 hr at 37°C. Remaining steps were performed as described above.

### Solid-phase and in-solution affinity selection

Phage-displayed naïve human antibody library comprising of 10 billion clones was employed for affinity selection. For in-solution affinity selection, 4 x 10^12^ phages from each of the eight phage sub-libraries were pooled, and the total of 3.2 x 10^13^ phages (~ 3200 fold excess of the library) in a volume of 1.6 ml was mixed with 1.6 ml 4% BSA-PBS. The phage library (3.2 ml) was divided into three tubes (2 ml, click cap, clear, round bottom microtubes, Treff Lab, Switzerland), and preadsorbed on MyOne streptavidin T1 beads (100 μl per tube, washed twice with 2% BSA-PBS) for 1 hr at 25°C on vertical rotator at 5 rpm for end-to-end mixing. After separation of streptavidin beads using magnetic particle concentrator, the preadsorbed library was collected in fresh tubes, and MTC28-Bio protein was added (at a final concentration of 100 nM per tube), followed by incubation for 2 hr at 25°C with rotation at 5 rpm. To each tube, 100 μl of pre-washed M280 streptavidin beads were added, followed by incubation for 30 min at 25°C with rotation at 5 rpm to capture biotinylated antigens along with bound phages. The beads were washed 10 times with PBST (1 x PBS with 0.05% Tween 20), and 10 times with 1 x PBS. Finally, the beads were incubated with 100 mM triethylamine (500 μl per tube) for 10 min at 25°C with rotation at 5 rpm, and phage eluate from 3 tubes was pooled. The eluate (total 1.5 ml) was neutralized with 750 μl 1 M Tris-HCl, pH 7.5 and an aliquot was used to determine the phage titer by infecting *E*. *coli* TOP10F’ cells. The remaining pool was used for infecting 20 ml *E*. *coli* TOP10F’ cells at 37°C for 30 min. After infection, the cells were harvested by centrifugation at 5000 rpm for 5 min at 25°C and re-suspended in 4 ml LB media. The cells were plated on 4 x 150 mm LBAmp_100_Glu_1%_ plates (LB media containing 100 μg/ml ampicillin and 1% glucose) to select for phage transductants. Following day, the cells from 150 mm LBAmp_100_Glu_1%_ plates were scraped in fresh 2 x YTGlu_1%_ media (2 x YT media containing 1% glucose), mixed with equal volume of 2 x glycerol storage solution (65% glycerol, 0.1 M Tris-HCl, pH 8.0, 25 mM MgSO_4_), and stored at -80°C. An aliquot of cells (~ 5 x 10^10^ cells) was used for rescue of phages using AGM13 helper phage in 1200 ml volume. The phages were harvested and purified using double PEG-NaCl precipitation [29], and used for the subsequent round of affinity-selection. In total, three rounds of affinity selection were performed using the same protocol as described for the first round with appropriate changes.

For passive immobilization-based solid-phase affinity selection, 16 immunotubes were coated with 40 μg of H10-MTC28 protein (10 μg/ml in 4 ml 1 x PBS) for 16 hr at 4°C on a vertical rotator at 10 rpm for end-to-end coating, followed by 1 hr incubation at 37°C. One tube was kept as no-coat control and contained only 1 x PBS. The tubes were washed thrice with 1 x PBS (no Tween-20), followed by blocking with 2% SM-PBS (2% Skimmed Milk-PBS) for 2 hr at 25°C with rotation at 10 rpm. Blocked immunotubes were washed thrice with 1 x PBS. Separately, approximately 2.4 x 10^13^ phages were diluted in 2% SM-PBS to 64 ml, and pre-adsorbed on skimmed milk-coated immunotubes tubes for 2 hr at 25°C to eliminate milk and plastic binders. Pre-adsorbed phages were added to H10-MTC28-coated tubes followed by incubation for 2 hr at 25°C with rotation at 10 rpm. The unbound phages were removed by aspiration, followed by 10 washes each with 4 ml of PBST (1 x PBS containing 0.05% Tween-20), and 5 washes with 1 x PBS. The bound phages were eluted using 1 ml of 100 mM triethylamine (prepared fresh in distilled water) per tube by rotation on test tube roller for 10 min at 25°C. The eluate was neutralized with 500 μl of 1 M Tris-HCl, pH 7.5. The titer of eluates from the coat and no-coat tubes and input phages was determined by infecting phages in *E*. *coli* TOP10F’ cells at appropriate dilutions. The eluate from the coated tube was processed as described for in-solution selection. In total, three rounds of selection were performed using the same protocol as described for the first round with appropriate changes.

## Results

### Construction of the vector pVMExp14367

**Fig 1:**
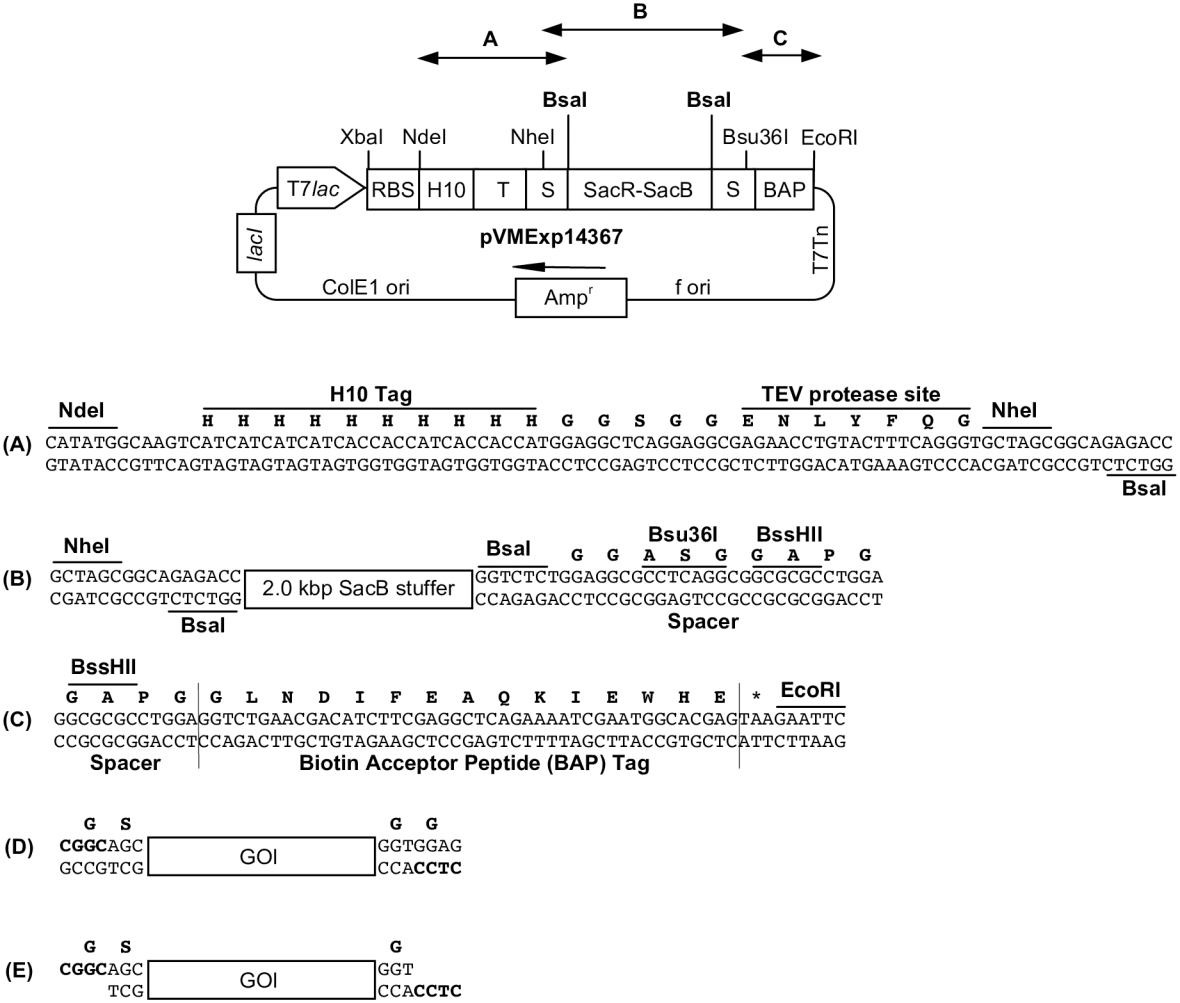
Diagrammatic representation of T7 promoter-lac operator-based pVMExp14367 expression vector to obtain recombinant proteins with N-terminal H10-TEV and C-terminal BAP tag. Only relevant genes and restriction sites are shown. The maps are not to scale. T7*lac*, T7 promoter-lac operator; RBS, ribosome-binding site; H10, deca-histidine tag; TEV, Tobacco Etch Virus protease cleavage site; S, glycine-serine rich spacers; SacR-SacB, 2.0 kbp SacR-SacB gene cassette flanked by BsaI sites; BAP, Biotin Acceptor Peptide; T7Tn, T7 transcription terminator; f ori, origin of replication of filamentous phage; Amp^r^, beta-lactamase gene; ColE1 ori, origin of replication. The amino acids encoded are shown in single letter code (bold) above the nucleotide sequence (A-D). (A-C) The sequence of the important components of vector including 2 BsaI cloning sites. (D) Sequence flanking the gene of interest (GOI) after PCR amplification. (E) GOI carrying 5’- 4 base overhangs generated after treatment with T4 DNA polymerase in the presence of dTTP.

A T7 promoter-lac operator-based IPTG/lactose inducible vector pVMExp14367 was employed for expression of recombinant proteins in *E*. *coli* carrying N-terminal deca-histidine tag (H10) followed by TEV protease site and C-terminal Biotin Acceptor Peptide (BAP) tag with appropriate glycine-serine rich spacers (Fig 1). This format allows for affinity-based purification of the expressed recombinant proteins using the H10 tag, and its subsequent specific removal by virtue of the TEV protease site to obtain proteins devoid of the N-terminal tag. The C-terminal BAP tag allows for *in vitro* site-specific biotinylation of the proteins using recombinant *E*. *coli* BirA enzyme. The vector design is compatible with highly efficient and high-throughput method for restriction enzyme-free cloning of the genes [27]. In this strategy, the vector is prepared by digestion with Type IIs restriction enzyme BsaI, whose two recognition sites are present in the vector flanking the stuffer in two orientations (Fig 1B) in a manner that allows generation of 4 base 5’-overhangs. The insert for cloning is prepared by PCR amplification using primers carrying 20–23 base long gene-specific sequence with 7 base long additional sequence (5’-CGGCACC-3’ in forward primer and 5’-CTCCACC-3’ in reverse primer) [27]. The resulting insert is then subjected to treatment with T4 DNA polymerase in the presence of dTTP to generate desired 4 base 5’-overhangs compatible with the vector (shown in bold; Fig 1D and 1E).

Five *M*. *tuberculosis* H37Rv secretory proteins, namely, MTC28 (Rv0040c), MPT63 (Rv1926c), MPT64 (Rv1980c), Ag85A (Rv3804c), and Ag85B (Rv1886c) were chosen as model proteins for this study. The DNA encoding these proteins (without signal sequence) was cloned in the vector pVMExp14367. Transformants in *E*. *coli* host BL21 (DE3) RIL were screened by sequencing and 100% cloning efficiency was observed (S1C Fig).

### Expression and localization of the proteins

Protein expression was performed using auto-induction process in ZYM5052 media [28] at low-temperature conditions that promote solubility of the proteins. All the five proteins showed good expression in total cell fractions (S2 Fig, Lane A), and constituted ~ 15–30% of the total cellular protein. However, the yield of soluble protein in the HHSS cytosolic fraction varied among different proteins (S2 Fig, Lane D). MTC28, MPT63, and MPT64 proteins were nearly 100% soluble, with almost the entire fraction of expressed protein present in the HHSS fraction, whereas the amount of Ag85A and Ag85B proteins in the HHSS fraction was only ~ 5% of the total expressed protein (S2 Fig, Lane D). Based on the yield of the soluble proteins, appropriate volumes of auto-induced cultures were processed (Table 1, Column 1), and the 2 x HHSS fraction containing soluble protein was subjected to purification.

**Table 1:** Summary of purification of five recombinant biotin-tagged proteins.

Protein (H10-T-POI-BAP)	Volume of culture (OD_600nm_) (1)	Amount of POI in HHSS^a^ (2)	Yield after Ni-affinity chromatography^b^^,^^c^ (3)	Yield (monomer) after gel filtration chromatography^d^^,^^c^ (4)	Yield after TEV protease removal^c^ (5)	Yield after desalting^f^^,^^c^ (6)	Yield after anion exchange chromatography^g^ (7)
MTC28	560 ml (9.8)	~ 330 mg/ 280 ml	~ 300 mg/ 24 ml	~ 200 mg^e^/ 36 ml	~ 180 mg/ 60 ml	~ 150 mg/ 80 ml	~ 112 mg/ 12 ml
MPT63	560 ml (8.9)	~ 330 mg/ 280 ml	~ 140 mg/ 26 ml	~ 190 mg/ 48 ml	~ 170 mg/ 69 ml	~ 150 mg/ 96 ml	~ 120 mg/ 12 ml
MPT64	560 ml (9.2)	~ 330 mg/ 280 ml	~ 190 mg/ 26 ml	~ 270 mg/ 44 ml	~ 255 mg/ 66 ml	~ 225 mg/ 88 ml	~ 150 mg/ 12 ml
Ag85A	2000 ml (11.1)	~ 100 mg/ 1000 ml	~ 90 mg/ 28 ml	~ 75 mg/ 40 ml	~ 75 mg/ 66 ml	~ 55 mg/ 83 ml	~ 41 mg/ 14 ml
Ag85B	2000 ml (9.5)	~ 120 mg/ 1000 ml	~ 90 mg/ 28 ml	~ 95 mg/ 44 ml	~ 90 mg/ 70 ml	~ 70 mg/ 88 ml	~ 42 mg/ 10 ml

^a^ The amount of recombinant protein (Protein of Interest; POI) was estimated based on analysis of Coomassie brilliant blue R-250 stained gel.

^b^ 20 ml NiFF column was employed for affinity chromatography.

^c^ The estimation may not be accurate due to high protein concentration and A_280nm_ measurement with AKTA flow cell.

^d^ Gel-filtration chromatography was performed on 480 ml column (XK 26/100; GE Healthcare). Superdex 75 resin was used for MTC28, Ag85A, and Ag85B proteins. Superdex 200 resin was used for MPT63 and MPT64 proteins.

^e^ MTC28 protein was dimeric.

^f^ Desalting was performed in two runs on 140 ml Sephadex G-25 (fine) column.

^g^ Ion-exchange chromatography was performed on 20 ml QHP anion-exchange column. Buffer system containing 20 mM Tris-HCl, pH 8.0 was used for MTC28, Ag85A, and Ag85B proteins. Buffer system containing 20 mM Tris-HCl, pH 7.5 was used for MPT63 and MPT64 proteins.

### Purification of the proteins

**Fig 2:**
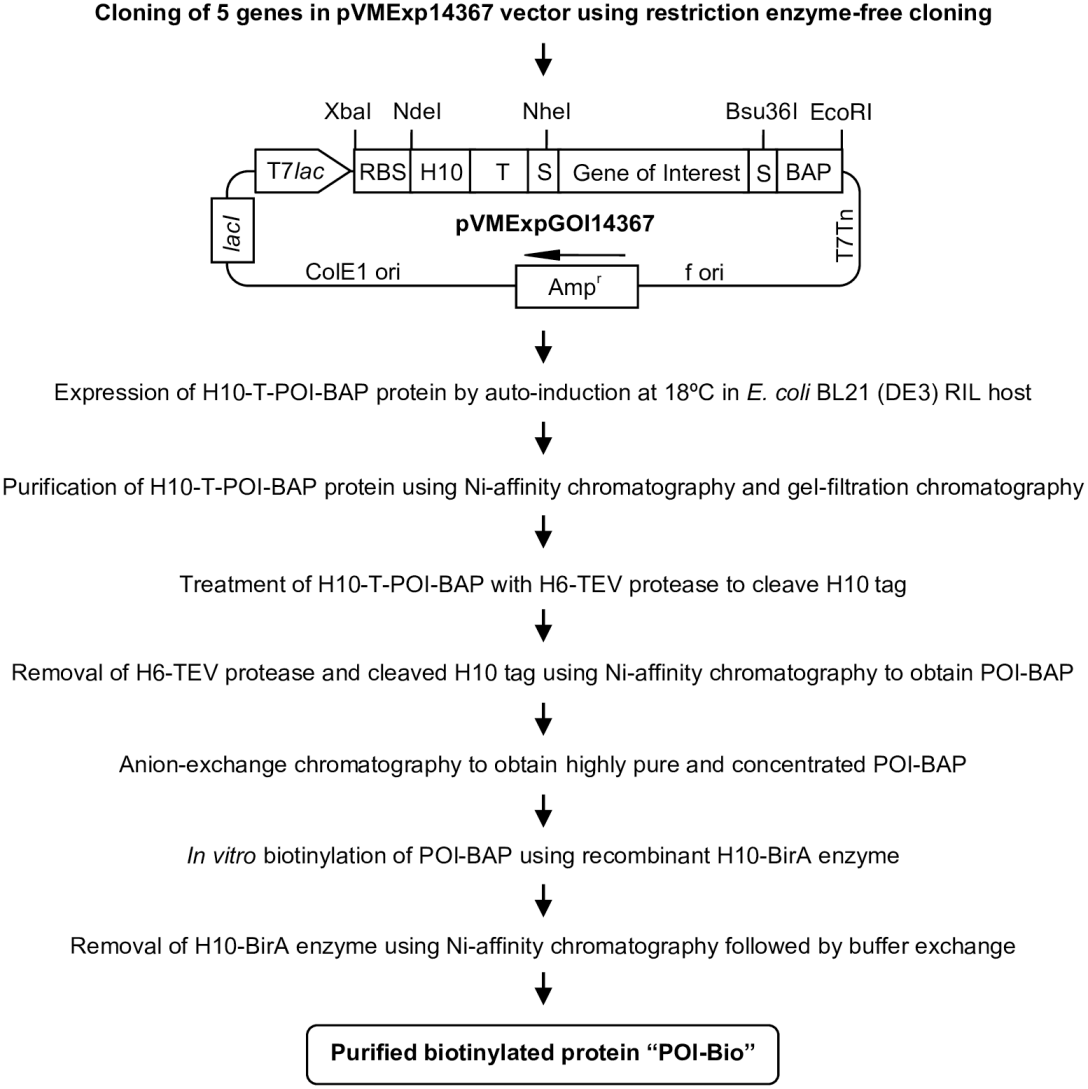
Workflow for expression, purification, and *in vitro* biotinylation of five mycobacterial proteins.

The recombinant proteins carried H10 tag followed by TEV protease site at the N-terminus, and BAP tag at the C-terminus (i.e., H10-T-POI-BAP; POI-Protein of interest). To purify proteins, a streamlined workflow was developed (Fig 2), which involved two-step purification including Ni-affinity chromatography and gel-filtration chromatography. The purified monomeric/dimeric fraction of the proteins obtained after gel-filtration chromatography was then subjected to treatment with H6-TEV protease to cleave the N-terminal H10 tag, followed by removal of TEV protease and cleaved tag. The proteins (devoid of H10 tag) were finally purified using anion-exchange chromatography and then subjected to *in vitro* biotinylation using H10-BirA enzyme and purification of the fully biotinylated protein. The purification of the representative protein MTC28 is described in detail.

**Fig 3:**
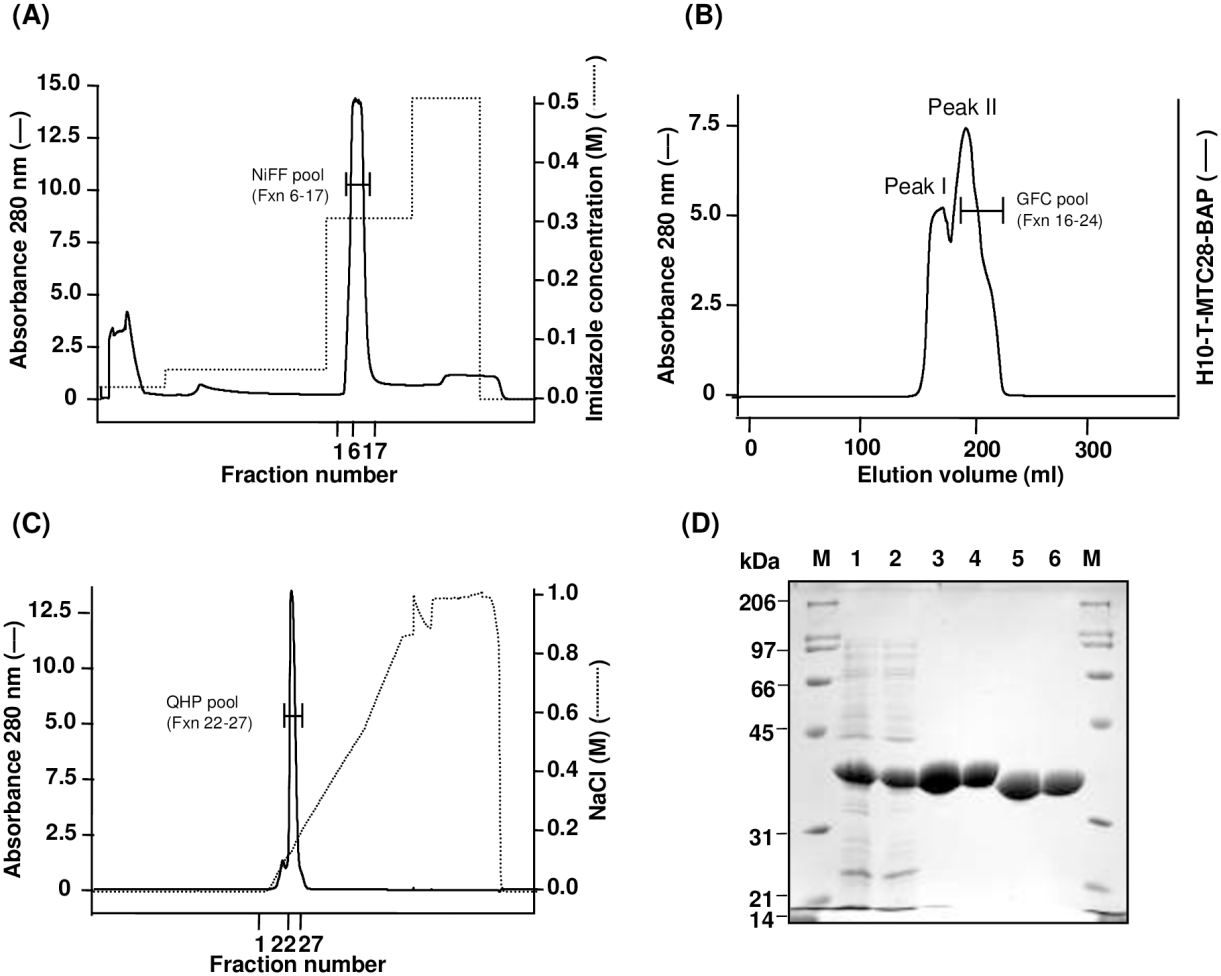
Purification of MTC28-BAP protein. Chromatogram showing (A) Elution profile of H10-T-MTC28-BAP protein on Ni Sepharose Fast Flow (NiFF) affinity column. Fraction numbers 6–17 were pooled (NiFF pool). (B) Elution profile of H10-T-MTC28-BAP protein on Superdex 75 gel-filtration column. Fraction numbers 16–24 were pooled (GFC pool). The GFC pool was treated with H6-TEV protease to cleave H10 tag from the protein followed by removal of cleaved tag and H6-TEV protease using Ni-affinity chromatography. (C) Elution profile of MTC28-BAP protein on Q Sepharose HP column. Fraction numbers 22–27 were pooled (QHP pool). (D) SDS-PAGE analysis of H10-T-MTC28-BAP protein at different stages during purification. The samples were analyzed by 0.1% SDS-12.5% PAGE under reducing conditions. The protein bands were visualized with Coomassie brilliant blue R-250 staining. Lane M, molecular weight marker, broad range (Bio-Rad, Hercules, CA) (shown in kDa); Lane 1, total cell after homogenization; Lane 2, High-High Speed Supernatant; Lane 3, NiFF pool; Lane 4, GFC pool; Lane 5, NiFF-TT pool (after desalting); Lane 6, QHP pool.

For the purification of H10-T-MTC28-BAP, HHSS containing approximately 330 mg of recombinant protein was applied on Ni Sepharose Fast Flow (NiFF) column in the presence of 20 mM imidazole to allow specific binding of the H10-tagged recombinant protein. The column was washed with 50 mM imidazole to remove loosely bound non-specific proteins followed by the elution of the desired protein in the presence of 300 mM imidazole (Fig 3A). Based on the SDS-PAGE analysis, the extent of purity was >90% in the NiFF pool, and it contained a small amount of both low and high molecular weight contaminants (Fig 3D, Lane 3). The NiFF pool was further subjected to gel-filtration chromatography on Superdex 75 column. The protein eluted between 0.3–0.5 CV in two peaks, which were not well-resolved (Fig 3B). Based on the elution volume of the peaks and that of gel-filtration calibration standards, it was estimated that peak I comprised of very high molecular weight proteins (possibly containing aggregated H10-T-MTC28-BAP or other high molecular weight contaminants), and peak II comprised of ~ 60–70 kDa molecular weight protein. The fractions comprising both the peaks were analyzed using SDS-PAGE under reducing conditions, and peak I was found to contain high molecular weight contaminants (which were also observed in NiFF pool), whereas later part of Peak II contained relatively pure H10-T-MTC28-BAP free of any visible contaminants and was pooled (GFC pool) for further processing (S3A Fig). Since, the molecular weight of the protein in peak II corresponded to ~ 60–70 kDa, we concluded that the H10-T-MTC28-BAP protein (molecular weight 34.2 kDa) was present as a dimer.

For the removal of H10 tag from purified H10-T-MTC28-BAP protein, the GFC pool was treated with H6-TEV protease (Table 1, Column 4). Following digestion, the reaction mixture was applied on NiFF column to remove H6-TEV protease, cleaved H10 tag, and uncleaved H10-T-MTC28-BAP protein (if any), and the desired MTC28-BAP protein was obtained in the flow-through (NiFF-TT pool). The MTC28-BAP protein in NiFF-TT pool was finally purified using anion-exchange chromatography on QHP column. The protein eluted as one major peak (Fig 3C) and contained highly pure protein (QHP pool; Fig 3D, Lane 6). Approximately 112 mg highly purified MTC28-BAP protein was obtained from 560 ml culture (Table 1, Column 7).

H10-T-MPT63-BAP, H10-T-MPT64-BAP, H10-T-Ag85A-BAP, and H10-T-Ag85B-BAP proteins were purified following a similar procedure as described for H10-T-MTC28-BAP protein. Based on the yield of proteins in the soluble fraction, different volumes of HHSS were processed for NiFF chromatography (Table 1, Columns 1, 2). For H10-T-MPT63-BAP, and H10-T-MPT64-BAP proteins, SDS-PAGE analysis after NiFF chromatography revealed that the extent of purity was >90%, and NiFF pool contained small amounts of both low and high molecular weight contaminants (S4D and S5D Figs, Lane 3). However, in the case of H10-T-Ag85A-BAP and H10-T-Ag85B-BAP proteins, a distinct band at approximately 60 kDa was observed as a major contaminant after NiFF chromatography (S6D and S7D Figs, Lane 3). This band could be a chaperone like GroEL [30], which is often found to be associated with unfolded/partially proteins. Since these two proteins, namely, Ag85A and Ag85B are sparingly soluble in *E*. *coli* cytosol (S2 Fig, Lane D), it is likely that these could be only partially folded and might have a tendency to remain associated with molecular chaperones. Thus, we incorporated a gel-filtration chromatography step in our purification pipeline to obtain a highly pure monomeric form of proteins. The NiFF pool of these proteins was purified using gel-filtration chromatography on Superdex 200 (for H10-T-MPT63-BAP and H10-T-MPT64-BAP proteins) or Superdex 75 (for H10-T-Ag85A-BAP and H10-T-Ag85B-BAP proteins) column. All four proteins eluted as two sharp and well-separated peaks (S4B–S7B Figs). For all the four proteins, SDS-PAGE analysis of peak I and peak II obtained in analytical-scale gel-filtration chromatography of the NiFF pool showed that peak I contained some high molecular weight contaminants, whereas Peak II contained pure form of the desired proteins free of any visible contaminants (S3B–S3F Fig). Based on the elution volume of the peaks and that of gel-filtration calibration standards, it was estimated that for all the four proteins, the peak II comprised of monomeric form of respective proteins and was pooled for further processing (GFC pool; S4D–S7D Figs, Lane 4). The GFC pools were treated with H6-TEV protease followed by its removal (as described before for H10-T-MTC28-BAP protein) to obtain proteins devoid of N-terminal H10 tag (Table 1, Columns 5, 6, and S4D–S7D Figs, Lane 5). These proteins were further purified using anion-exchange chromatography on QHP column. All the four proteins showed elution profile with one major peak (S4C–S7C Figs). SDS-PAGE analysis showed that fraction number 27–32 for MPT63-BAP, 24–29 for MPT64-BAP, 31–37 for Ag85A-BAP, and 31–35 for Ag85B-BAP contained highly pure proteins, and were pooled (QHP pool, S4D–S7D Figs, Lane 6). The final yield of each purified protein is summarized in Table 1, Column 7.

### *In vitro* biotinylation of the purified proteins using H10-BirA enzyme

To perform biotinylation of the purified C-terminal BAP-tagged proteins, the QHP pool was subjected to *in vitro* biotinylation using H10-BirA enzyme in the presence of biotin and ATP. For this, the proteins were desalted in the appropriate buffer, and treated with H10-BirA. Following biotinylation, the H10-BirA enzyme was removed using NiFF column and the flow-through fraction containing biotinylated protein was desalted. The SDS-PAGE analysis revealed that integrity of proteins was maintained during biotinylation process (S8 Fig).

### Determination of the extent of biotinylation in biotin-tagged proteins

As a measure of the efficiency of the *in vitro* biotinylation reaction, the extent of biotinylation in biotin-tagged proteins was determined. As described in methods, a fixed amount of biotin-tagged proteins was adsorbed on Streptavidin Sepharose HP resin, and total immunoreactive protein was estimated in ‘before’ and ‘after’ adsorption fractions using indirect ELISA on Nunc Immobilizer streptavidin-coated plates by protein-specific monoclonal antibodies. Based on the fold reduction in the reactivity of fractions after adsorption on streptavidin beads (a measure of remaining protein amount), the extent of biotinylation was determined and was found to be greater than 99.5% for all the five biotin-tagged proteins (Table 2).

**Table 2:** Determination of the extent of biotinylation of proteins after *in vitro* biotinylation using recombinant H10-BirA enzyme.

Protein	Fold dilution to achieve A_450nm_ ~ 0.5 ^a^		Fold reduction in protein reactivity after adsorption	Extent of biotinylation
	Before adsorption on streptavidin beads	After adsorption on streptavidin beads		
MTC28-Bio	25,000	50	500	99.8%
MPT63-Bio	100,000	100	1000	99.9%
MPT64-Bio	25,000	50	500	99.8%
Ag85A-Bio	20,000	5	4000	99.9%
Ag85B-Bio	30,000	30	1000	99.9%

Fixed amount of biotin-tagged proteins was adsorbed on Streptavidin Sepharose HP beads. This was followed by estimation of protein amount in ‘before’ and ‘after’ adsorption fractions using indirect ELISA on Nunc Immobilizer streptavidin-coated plates, where the proteins were probed with specific monoclonal antibodies followed by detection using HRP-conjugated Goat anti-Mouse IgG (H+L) antibody. The values are based on the mean of two independent experiments.

^**a**^ The number of times fraction was diluted to obtain A_450nm_ of approximately 0.5 in indirect ELISA.

### Biotin-tagged proteins are superior coating molecules for ELISA

Recombinant immunodominant proteins of infectious organisms are routinely employed in ELISA for the detection of disease-specific antibodies in the body fluids of suspected patients [5, 6, 17, 31–33]. However, immobilization of these proteins on the surface of ELISA plates can be variable and inefficient [11]. We decided to evaluate the performance of biotin-tagged proteins in ELISA using immobilization by passive adsorption on the polystyrene surface, and specific capture on the streptavidin-coated surface. For this, different concentrations of biotin-tagged proteins were coated on the two types of microtiter plate surfaces, and the captured proteins were detected using indirect ELISA with either specific mouse monoclonal antibodies, or rabbit polyclonal antibodies. In this assay, the amount of protein added for coating on the two surfaces to produce equal reactivity was compared.

**Fig 4:**
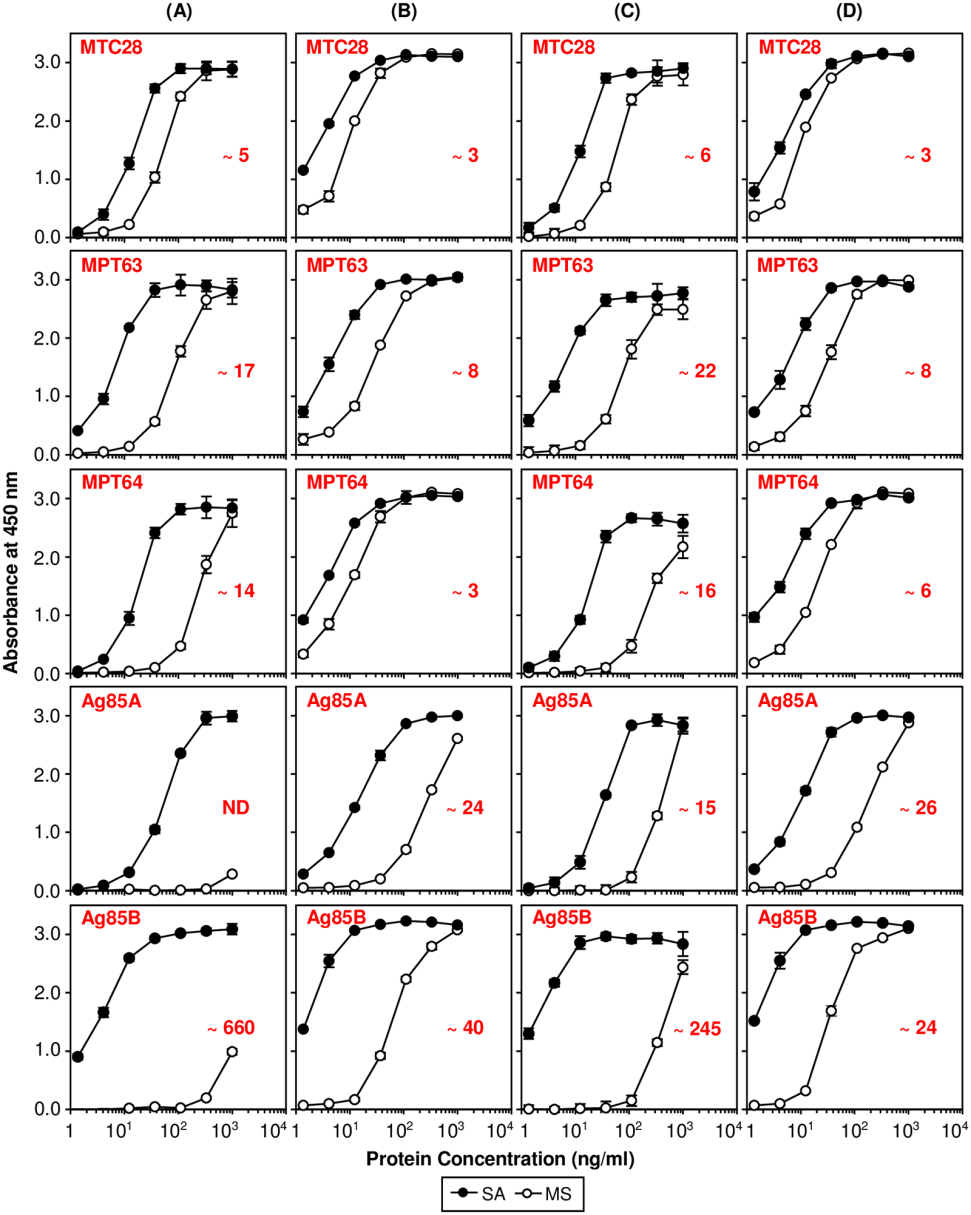
Comparison of the ELISA reactivity of proteins at different coating concentrations upon passive and specific immobilization. The five biotin-tagged proteins were immobilized individually or as a mixture (equal, w/w) on Nunc Immobilizer streptavidin-coated (SA) or Nunc Maxisorp (MS) plates at different concentrations. The bound protein was probed with specific mouse monoclonal (MAb) or rabbit polyclonal antibodies (PAb), followed by detection using HRP-conjugated Goat anti-Mouse IgG (H+L) antibody or Goat anti-Rabbit IgG (H+L) antibody. (A) Detection of five biotin-tagged proteins immobilized individually on SA or MS plates with MAb. (B) Detection of five biotin-tagged proteins immobilized individually on SA or MS plates with PAb. (C) Detection of five biotin-tagged proteins immobilized as a mixture on SA or MS plates with MAb. (D) Detection of five biotin-tagged proteins immobilized as a mixture on SA or MS plates with PAb. Identity of the protein and the approximate fold change in the amount of protein required to produce equal reactivity (calculated as the ratio of absorbance readings from the linear region of the graphs of specific versus passive immobilization) is indicated inside the graph panel in red color. The protein concentration values on X-axis for (C) and (D) correspond to the concentration of individual proteins in the mixture. ELISA values shown are mean ± SD of three independent experiments. ND, Not Determined.

When detected with monoclonal antibodies, the assay required approximately 5–660 fold less protein to produce comparable signal in ELISA upon specific capture using streptavidin-coated plates, which is due to the efficient capture of biotin-tagged proteins on the streptavidin-coated surface (Fig 4A). This was especially evident in the case of Ag85A-Bio and Ag85B-Bio proteins, where for passive adsorption, much higher protein concentration (greater than 1000 ng/ml) was required to produce a significant signal (A_450nm_ of ~ 1.0) in comparison to MTC28-Bio, MPT63-Bio, and MPT64-Bio proteins, which were required in much lower concentrations (approximately 100–200 ng/ml) (Fig 4A). This suggested that Ag85A-Bio and Ag85B-Bio proteins tend to coat poorly on the polystyrene surface during passive adsorption. However, upon specific capture on the streptavidin-coated plates, both Ag85A-Bio and Ag85B-Bio proteins produced a significant signal at lower protein concentrations (approximately 1–30 ng/ml), which was comparable to other biotin-tagged proteins (approximately 1–10 ng/ml) (Fig 4A).

When the detection in ELISA was performed using polyclonal antibodies, efficient protein-coating was observed upon specific capture on the streptavidin-coated plates (Fig 4B). However, the difference between specific and passive immobilization was less pronounced (approximately 3–40 fold) here as compared to the assay performed using monoclonal antibodies (Fig 4A versus 4B). This difference could likely be due to the higher affinity and polyclonal nature of the rabbit antibodies [34–36]. The monoclonal antibodies bind to only one site on the protein (the epitope), and the same may not be accessible on every passively coated molecule, whereas, the polyclonal antibodies bind to multiple epitopes on the same molecule, and thus, even upon passive coating, a larger number of epitopes could be accessible. In contrast, on streptavidin-coated plates, all the epitopes are likely to be exposed, except for those, which are present at the C-terminus of the protein; therefore, even monoclonal antibodies show significant binding.

To mimic the assay conditions, where the coating of proteins as a mixture may be necessary, we also compared the efficiency of specific and passive immobilization of each protein in a mixture. In this case also, the specific immobilization resulted in reduced requirement of proteins to produce comparable reactivity in ELISA upon detection both mouse monoclonal antibodies (~ 6–245 fold), and rabbit polyclonal antibodies (~ 3–26 fold) (Fig 4C and 4D).

This property of efficient capture of biotinylated proteins using streptavidin-coated plates can be used as an effective strategy to improve the ELISA sensitivity, particularly for the proteins like Ag85A and Ag85B that exhibit poor coating characteristics upon passive immobilization. This finding is particularly relevant for developing ELISA-based assays for antibody detection, where it might be necessary to coat multiple proteins with variable surface binding characteristics.

### Characterization of biotin-tagged proteins using phage display-based affinity selection

We compared the efficiency for isolating specific antibody binders from a phage-displayed naive human antibody library (10 billion clones in scFv format) against recombinant H10-MTC28 protein by its passive immobilization on Nunc immunotubes or against biotin-tagged MTC28-Bio protein by its specific capture on streptavidin-coated M280 magnetic beads. Three rounds of solid-phase affinity selection were performed on H10-MTC28 protein coated passively on immunotubes at 10 μg/ml, and 48 transductants were screened by phage ELISA. All 48 clones (100%) showed reactivity to MTC28 protein indicating enrichment of specific binders (Table 3). Similarly, three rounds of in-solution affinity selection were performed on biotin-tagged MTC28-Bio at 100 nM concentration (~ 3 μg/ml), and 24 transductants were screened after the third round of selection. All 24 clones (100%) were found to be reactive to the target protein (Table 3). Though the efficiency of the two selection procedures in terms of yielding positive clones after the third round of affinity selection was same, it is worth noting that in-solution selection led to the higher recovery of phages during selection (Table 3, output/input phage ratio). This could have been due to a higher degree of non-specific selection of phages during in-solution selection, but screening of transductants even after a second round of in-solution selection revealed that ~ 75% clones were reactive to the target protein. This confirmed that the higher phage recovery numbers are not due to non-specific enrichment of phages, but should be due to improved availability of the bait. Most importantly, during in-solution affinity selections, the concentration of bait can be carefully controlled and usually reduced in subsequent rounds of selection to obtain binders with higher affinities. In addition, the amount of protein required for specific capture-based in-solution affinity selection was much lower (less than 50 micrograms) as compared to the amount required for passive immobilization-based solid-phase affinity selection (few milligrams).

**Table 3:** Summary of three rounds of solid-phase and in-solution affinity selection of human naïve scFv library on MTC28 antigen.

H10-MTC28 (Solid-phase; Immunotubes^a^)				MTC28-Bio (in-solution; Streptavidin beads^b^)			
Round	I	II	III	Round	I	II	III
**Antigen coating concentration**	10 μg/ml	10 μg/ml	10 μg/ml	**Antigen concentration**	100 nM	100 nM	100 nM
**No. of tubes coated**	16	8	6	**Total Binding Reaction Volume**	3.2 ml	3 ml	3 ml
**No. of washes** **(PBST + PBS)**	10 + 5	10 + 5	15 + 10	**No. of washes** **(PBST + PBS)**	10 + 10	10 + 10	15 + 15
**Total input phage**	2.4 x 10^13^	2 x 10^13^	1 x 10^13^	**Total input phage**	3.2 x 10^13^	3 x 10^12^	6 x 10^11^
**Total output phage**	4.8 x 10^6^	7.2 x 10^6^	5.4 x 10^8^	**Total output phage**	5.4 x 10^7^	6.3 x 10^7^	4.5 x 10^8^
**Output/Input phage ratio**	2 x 10^−7^	3.6 x 10^−7^	5.4 x 10^−5^	**Output/Input phage ratio**	1.6 x 10^−6^	2.1 x 10^−5^	7.5 x 10^−4^
**No. of clones analyzed in ELISA**	ND	ND	48	**No. of clones analyzed in ELISA**	ND	20	24
**Positive clones based on ELISA**	ND	ND	48 (100%)	**Positive clones based on ELISA**	ND	15 (75%)	24 (100%)

^a^ Nunc Maxisorp Polystyrene Immunotubes, 12 x 75 mm were used for passive protein coating for solid-phase affinity selection.

^b^ Streptavidin M-280 Dynabeads were used for specific capture of biotin-tagged protein for in-solution affinity selection.

ND: Not Determined

## Discussion

In this paper, we have described a streamlined pipeline for easy and robust cloning, high-level cytosolic expression, purification, and *in vitro* biotinylation to produce biotin-tagged proteins (Fig 2). The study also demonstrates applications of biotin-tagged proteins in multiplexed ELISA and affinity selection using a phage-displayed naïve human antibody library. The results strongly indicate that the method of protein immobilization is an important determinant of the indirect ELISA sensitivity and the efficiency of phage display-based affinity selection.

The expression vector pVMExp14367 is based on a well-established restriction enzyme-free cloning strategy developed in our laboratory (Fig 1) [27]. The vector employs an IPTG/lactose inducible T7 promoter-lac operator system to drive the high-level cytosolic expression of proteins carrying N-terminal H10 tag with TEV protease site and C-terminal BAP tag (H10-T-POI-BAP). The recombinant proteins were expressed using lactose-based auto-induction and were purified from the soluble cytosolic fraction using a combination of affinity, gel-filtration, and ion-exchange chromatography integrated with a well-optimized step for the removal of deca-histidine tag from the recombinant using H6-TEV protease to yield POI-BAP (Fig 2). In the purification strategy, while the affinity chromatography step allows initial purification of the recombinant H10-T-POI-BAP proteins, the subsequent gel-filtration step allows the separation of large aggregated proteins or low/high molecular weight contaminants from the monomeric / properly folded proteins and leads to buffer-exchange making the purified protein preparation compatible for TEV protease treatment. In our vector design, for better accessibility, the TEV protease cleavage site is placed between the deca-histidine tag and the POI with appropriate spacer sequences. For this reason, the H10 tag of all the five proteins could be cleaved efficiently by recombinant H6-TEV protease to yield proteins carrying only BAP tag at C-terminus (POI-BAP). It should be noted that after the removal of deca-histidine tag, the POI-BAP preparations are highly pure and can be directly used for several applications. However, we have included an additional ion-exchange-based chromatography step to further purify and obtain concentrated protein preparations.

The purified POI-BAP preparations carrying BAP tag at C-terminus were subjected to *in vitro* biotinylation using recombinant H10-BirA enzyme. Several systems have been described for co-expression of BirA enzyme that lead to *in vivo* biotinylation of the over-expressed recombinant proteins [13, 37, 38]. However, it may often require optimization of the expression levels of the recombinant protein and BirA enzyme to obtain sufficiently biotinylated protein [18]. This may be particularly troublesome while dealing with multiple proteins, where different proteins can show variable levels of biotinylation [13]. *In vivo* biotinylation may also be difficult while dealing with insoluble proteins, which may go into inclusion bodies, thereby making BAP tag inaccessible [18]. In addition, BirA enzyme when expressed at high levels tends to become insoluble and requires expression with solubility enhancers such as MBP or thioredoxin [39]. Furthermore, we have observed that during *in vivo* biotinylation, over-expressed BirA enzyme remains attached to a significant fraction of biotin-tagged proteins, requiring additional step of purification. A recent report also recommends inclusion of biotin in the wash buffer to prevent the co-purification of BirA enzyme during purification of *in vivo* biotinylated proteins [40]. For these reasons, we preferred the *in vitro* biotinylation of the purified proteins over *in vivo* biotinylation. In addition, *in vitro* biotinylation led to near 100% biotinylation of proteins (Table 2). However, in some proteins, the C-terminal BAP tag may not be accessible leading to reduced biotinylation efficiency. Ikonomova et al. reported that the immobilization efficiency of the *in vivo* biotinylated scFvs carrying BCCP tag (87 amino acids) was superior to those carrying BAP tag (15 amino acids) at the C-terminus, suggesting the importance of the accessibility of the biotinylation site for BirA enzyme as well as accessibility of biotin moiety on the protein to bind to streptavidin [41]. To address this, our vector design incorporates a spacer sequence between the encoded POI and BAP tag for its improved accessibility to BirA enzyme. Another alternative approach could be a fusion of the BAP tag at the N-terminus of the protein if the C-terminal sequences of the POI are functionally important [18].

The model proteins chosen in this study for the development of protein purification and biotinylation workflows belong to *M*. *tuberculosis* H37Rv and have been shown to have immunodiagnostic potential as antibodies against these proteins are found in the sera of TB patients [14, 16, 17]. Therefore, as one of the prime application of the biotin-tagged proteins, we evaluated their use in indirect ELISA in two formats. In the first format, individual proteins were coated directly on polystyrene plates (by passive adsorption) or captured specifically on streptavidin-coated plates. The results showed that on the streptavidin-coated surface, much lower protein concentrations were required to produce an equivalent signal in ELISA as compared to passive adsorption (Fig 4A and 4B). Another important finding was that on streptavidin-coated plates, every protein was captured with almost equal efficiency, while in passive adsorption, every protein showed different coating behavior. In this study, this phenomenon was clearly observed with two proteins, namely, Ag85A-Bio and Ag85B-Bio, which showed very poor coating upon passive adsorption, but could be captured efficiently on the streptavidin-coated surface (Fig 4A and 4B). The results were consistent with both protein-specific mouse monoclonal and rabbit polyclonal antibodies (Fig 4A and 4B). Gross et al. reported similar findings, where a striking difference of 100–300 fold was observed in the ELISA sensitivity between streptavidin-based specific capture versus passive coating of erythropoietin (EPO) during detection of anti-EPO antibodies in human sera samples [42]. Batra et al. also demonstrated improved sensitivity during detection of anti-Dengue virus antibodies using biotinylated antigen [5]. Clavijo et al. also employed biotinylated recombinant protein for development of a competition ELISA-based test for the detection of foot-and-mouth disease virus antibodies [43].

In addition, there can be several immunoassay formats (ELISA, lateral flow etc.) that involve coating with a mixture of proteins on the relevant surface for the detection of specific antibodies in samples. This has been found to be more pertinent in the case of development of antibody detection-based assays for tuberculosis diagnostics, where testing against multiple proteins seems to be necessary to achieve desired range of sensitivity [14, 16, 17]. Since, the five biotin-tagged proteins employed in this study are well-suited for this application, we evaluated the efficiency of passive and specific immobilization of each protein when present in a mixture by testing their capture using mouse monoclonal or rabbit polyclonal antibodies. It was found that even in this situation, streptavidin-coated surface allowed efficient coating of every protein present in the mixture, irrespective of the nature of the detector antibody further underscoring the broad utility of this concept (Fig 4C and 4D). Thus, the specific capture of a mixture of biotin-tagged proteins on streptavidin surface opens avenues for multiplexing of proteins, especially in ELISA by allowing efficient and equal capture of the proteins in the mixture required for improved sensitivity.

In the literature, multi-epitope proteins or a fusion of multiple proteins have been described to suffice the requirement of multiplexing to achieve desired assay sensitivity or to detect many serotypes [5, 15, 44]. However, such proteins are difficult to produce due to solubility issues [5, 15, 44]. Biotin-tagged recombinant proteins will also find use in lateral flow strips where the capture proteins could be sprayed on the streptavidin-coated surface rather than being passively adsorbed on the membrane, which may also allow directional immobilization with higher protein density.

The findings of this work also suggest that the efficient capture of biotin-tagged proteins on streptavidin-coated surfaces would reduce the total amount of protein required, thus making the assays economical. Staudt et al. exploited the superior immobilization efficiency of the biotin-tagged proteins on the streptavidin-coated surface to develop antigen microarrays in 96 well plate format for high-throughput screening of hybridoma supernatants against several antigens in parallel [45]. This reduced the overall requirement of the proteins (for coating), and hybridoma supernatants during the assay making it more convenient and economical.

The results reported here also demonstrate improvement in the efficiency of isolating binders from a phage-displayed naïve human antibody library using biotin-tagged proteins as the bait. In-solution affinity selection showed improvement in the yield of specific phages by 1–2 orders of magnitude (Table 3). Overall, the specific capture-based in-solution affinity selection required much less amount of protein (less than 50 micrograms) as compared to the amount required for passive immobilization-based solid-phase affinity selection (few milligrams). Moreover, technically, because of the higher efficiency and ease of handling, it is easier to adapt the affinity selection method based on the use of streptavidin beads in a high-throughput format. Several groups have reported the successful use of biotinylated proteins as bait for the isolation of monoclonal antibodies from phage-displayed antibody libraries [7, 8, 13]. Routine panning protocols that involve passive adsorption of proteins on polystyrene surface may yield binders that recognize only denatured form of the target protein, which may not be desirable [7, 13]. Use of biotinylated proteins captured on streptavidin-coated plates or beads as bait can alleviate this problem [13]. Haque et al. have described an in-solution panning strategy employing *in vivo* biotinylated target antigen as a bait to select binders against a specific conformation of the target [7].

For some applications, especially those employing monoclonal antibodies, use of a biotinylated protein carrying BAP tag at the C-terminus might block reaction with antibodies requiring free C-terminus of the protein for their binding. Literature suggests that BAP tag can be attached at both N and C-terminus of the protein [13, 18]. Therefore, depending on the downstream application, the position of BAP tag can be decided.

In summary, the streamlined pipeline with efficient and robust protocols described here will accelerate the production of biotinylated proteins to facilitate several applications including the development of multiplexed immunoassays for antibody detection in patient sera, and selection of high-affinity phage-displayed antibodies.

## Supporting information

S1 Fig Details for cloning five genes of *M*. *tuberculosis* H37Rv in expression vector pVMExp14367. (A) Details of the template DNA and oligonucleotides used for the amplification of five genes. Oligonucleotide sequence in black bold letters denotes the 7 base tail appended on either ends of the gene specific sequence for cloning genes using restriction enzyme-free cloning method. (B) Agarose gel-based analysis of purified PCR products and digested vector. Lane M, Marker; Lane 1, Ag85A; Lane 2, Ag85B; Lane E, Extra gene not pursued in this study; Lane 3, MPT63; Lane 4, MPT64; Lane 5, MTC28; Lane 6, BsaI digested pVMExp14367 vector. (C) Summary of cloning efficiency of five genes. ^a^ The efficiency of electrocompetent *E*. *coli* BL21 (DE3) RIL cells was ~ 5 x 10^8^ per μg pGEM DNA.
(PDF)

S2 Fig Analysis of sub-cellular localization of five recombinant proteins of *M*. *tuberculosis* H37Rv expressed in *E*. *coli* BL21 (DE3) RIL host. Different sub-cellular fractions were analyzed using 0.1% SDS—8–20% gradient PAGE followed by visualization with coomassie brilliant blue R-250 stain. A, Total cell fraction; B, Total cell fraction after sonication; C, High Speed Supernatant (HSS); D, High-High Speed Supernatant (HHSS).
(PDF)

S3 Fig SDS-PAGE analysis of fractions constituting the peaks obtained during gel-filtration chromatography. To identify fractions containing desired protein without major contaminants after gel-filtration chromatography, fractions from preparative-scale gel-filtration chromatography (H10-T-MTC28-BAP) or analytical gel-filtration chromatography (other four proteins) were analyzed using SDS-PAGE under reducing conditions, and visualized with Coomassie brilliant blue R-250 staining. For H10-T-MTC28-BAP protein, gel-filtration chromatography of NiFF pool was performed on 480 ml Superdex 75 column (XK 26/100, GE Healthcare). For other proteins, analytical-scale gel-filtration chromatography of 2 ml NiFF pool was performed on 45 ml column (Tricorn 10/600, GE Healthcare) packed with Superdex 75 (H10-T-Ag85A-BAP and H10-T-Ag85B-BAP proteins) or Superdex 200 (H10-T-MPT63-BAP and H10-T-MPT64-BAP proteins), and 1 ml fractions were collected. (A) SDS-PAGE analysis of fractions obtained after gel-filtration chromatography of NiFF pool of H10-T-MTC28-BAP protein. Lane 1, NiFF pool; Lane 2–6, fraction number 4, 6, 8, 10, and 12 constituting peak I; Lane 7–13, fraction number 14, 16, 18, 19, 21, 23, and 24 constituting peak II. (B-F) SDS-PAGE analysis of fractions obtained after analytical gel-filtration chromatography of NiFF pool of other four proteins. (B) H10-T-MPT63-BAP protein (peak I). Lane 1, NiFF pool; Lane 2–12, fraction number 8–18. (C) H10-T-MPT63-BAP protein (peak II). Lane 1–8, fraction number 19–26. (D) H10-T-MPT64-BAP protein. Lane 1, NiFF pool; Lane 2–7, fraction number 7–12 constituting peak I; Lane 8–13, fraction number 19–24 constituting peak II. (E) H10-T-Ag85A-BAP protein. Lane 1, NiFF pool; Lane 2–5, fraction number 4–7 constituting peak I; Lane 6–12, fraction number 8–14 constituting peak II. (F) H10-T-Ag85B-BAP protein. Lane 1, NiFF pool; Lane 2–5, fraction number 4–7 constituting peak I; Lane 6–12, fraction number 8–14 constituting peak II. For all gels, Lane M denotes molecular weight marker in kDa (Broad range, Bio-Rad, Hercules, CA).
(PDF)

S4 Fig Purification of MPT63-BAP protein. Chromatogram showing (A) Elution profile of H10-T-MPT63-BAP protein on Ni Sepharose Fast Flow (NiFF) affinity column. Fraction numbers 6–18 were pooled (NiFF pool). (B) Elution profile of NiFF pool of H10-T-MPT63-BAP protein on Superdex 200 gel-filtration column. Fraction numbers 44–55 were pooled (GFC pool). The GFC pool was treated with H6-TEV protease to cleave H10 tag from the protein followed by removal of cleaved tag and H6-TEV protease using Ni-affinity chromatography. (C) Elution profile of MPT63-BAP protein on Q Sepharose HP column. Fraction numbers 27–32 were pooled (QHP pool). (D) SDS-PAGE analysis of H10-T-MPT63-BAP protein at different stages during purification. The samples were analyzed by 0.1% SDS-15% PAGE under reducing conditions. The protein bands were visualized with coomassie brilliant blue R-250 staining. Lane M, molecular weight marker, broad range (Bio-Rad, Hercules, CA) (shown in kDa); Lane 1, total cell after homogenization; Lane 2, High-High Speed Supernatant; Lane 3, NiFF pool; Lane 4, GFC pool; Lane 5, NiFF-TT pool (after desalting); Lane 6, QHP pool.
(PDF)

S5 Fig Purification of MPT64-BAP protein. Chromatogram showing (A) Elution profile of H10-T-MPT64-BAP protein on Ni Sepharose Fast Flow (NiFF) affinity column. Fraction numbers 6–18 were pooled (NiFF pool). (B) Elution profile of NiFF pool of H10-T-MPT64-BAP protein on Superdex 200 gel-filtration column. Fraction numbers 45–55 were pooled (GFC pool). The GFC pool was treated with H6-TEV protease to cleave H10 tag from the protein followed by removal of cleaved tag and TEV protease using Ni-affinity chromatography. (C) Elution profile of MPT64-BAP protein on Q Sepharose HP column. Fraction numbers 24–29 were pooled (QHP pool). (D) SDS-PAGE analysis of H10-T-MPT64-BAP protein at different stages during purification. The samples were analyzed by 0.1% SDS-12.5% PAGE under reducing conditions. The protein bands were visualized with coomassie brilliant blue R-250 staining. Lane M, molecular weight marker, broad range (Bio-Rad, Hercules, CA) (shown in kDa); Lane 1, total cell after homogenization; Lane 2, High-High Speed Supernatant; Lane 3, NiFF pool; Lane 4, GFC pool; Lane 5, NiFF-TT pool (after desalting); Lane 6, QHP pool.
(PDF)

S6 Fig Purification of Ag85A-BAP protein. Chromatogram showing (A) Elution profile of H10-T-Ag85A-BAP protein on Ni Sepharose Fast Flow (NiFF) affinity column. Fraction numbers 7–20 were pooled (NiFF pool). (B) Elution profile of NiFF pool of H10-T-Ag85A-BAP protein on Superdex 75 gel-filtration column. Fraction numbers 15–24 were pooled (GFC pool). The GFC pool was treated with H6-TEV protease to cleave H10 tag from the protein followed by removal of cleaved tag and TEV protease using Ni-affinity chromatography. (C) Elution profile of Ag85A-BAP protein on Q Sepharose HP column. Fraction numbers 31–37 were pooled (QHP pool). (D) SDS-PAGE analysis of H10-T-Ag85A-BAP protein at different stages during purification. The samples were analyzed by 0.1% SDS-12.5% PAGE under reducing conditions and visualized with coomassie brilliant blue R-250 staining. Lane M, molecular weight marker, broad range (Bio-Rad, Hercules, CA) (shown in kDa); Lane 1, total cell after homogenization; Lane 2, High-High Speed Supernatant; Lane 3, NiFF pool; Lane 4, GFC pool; Lane 5, NiFF-TT pool (after desalting); Lane 6, QHP pool.
(PDF)

S7 Fig Purification of Ag85B-BAP protein. Chromatogram showing (A) Elution profile of H10-T-Ag85B-BAP protein on Ni Sepharose Fast Flow (NiFF) affinity column. Fraction numbers 5–18 were pooled (NiFF pool). (B) Elution profile of NiFF pool of H10-T- Ag85B-BAP protein on Superdex 75 gel-filtration column. Fraction numbers 19–29 were pooled (GFC pool). The GFC pool was treated with H6-TEV protease to cleave H10 tag from the protein followed by removal of cleaved tag and TEV protease using Ni-affinity chromatography. (C) Elution profile of Ag85B-BAP protein on Q Sepharose HP column. Fraction numbers 31–35 were pooled (QHP pool). (D) SDS-PAGE analysis of H10-T-Ag85B-BAP protein at different stages during purification. The samples were analyzed by 0.1% SDS-12.5% PAGE under reducing conditions. The protein bands were visualized with coomassie brilliant blue R-250 staining. Lane M, molecular weight marker, broad range (Bio-Rad, Hercules, CA) (shown in kDa); Lane 1, total cell after homogenization; Lane 2, High-High Speed Supernatant; Lane 3, NiFF pool; Lane 4, GFC pool; Lane 5, NiFF-TT pool (after desalting); Lane 6, QHP pool.
(PDF)

S8 Fig SDS-PAGE analysis of five purified biotinylated proteins. After *in vitro* biotinylation and removal of H10-BirA enzyme, five microgram of each biotinylated protein was analyzed on 0.1% SDS—12.5% PAGE followed by visualization with Coomassie brilliant blue R-250 staining. Lane M, molecular weight marker, broad range (Bio-Rad, Hercules, CA) (shown in kDa); Lane 1, MTC28-Bio; Lane 2, MPT63-Bio; Lane 3, MPT64-Bio; Lane 4, Ag85A-Bio; Lane 5, Ag85B-Bio.
(PDF)
