# Supplementary material for: Biotin-tagged proteins: Reagents for efficient ELISA-based serodiagnosis and phage display-based affinity selection
Source: PLoS One. 2018 Jan 23;13(1):e0191315. doi: 10.1371/journal.pone.0191315 (PMC5779676; doi:10.1371/journal.pone.0191315)
Supplement: S1 Fig — (A) Details of the template DNA and oligonucleotides used for the amplification of five genes. Oligonucleotide sequence in black bold letters denotes the 7 base tail appended on either ends of the gene specific sequence for cloning genes using restriction enzyme-free cloning method. (B) Agarose gel-based analysis of purified PCR products and digested vector. Lane M, Marker; Lane 1, Ag85A; Lane 2, Ag85B; Lane E, Extra gene not pursued in this study; Lane 3, MPT63; Lane 4, MPT64; Lane 5, MTC28; Lane 6, BsaI digested pVMExp14367 vector. (C) Summary of cloning efficiency of five genes. a The efficiency of electrocompetent E. coli BL21 (DE3) RIL cells was ~ 5 x 10^8^ per μg pGEM DNA. (PDF) [file pone.0191315.s001.pdf]

(A)

Gene of Interest	Size (bp)	Template for PCR	Primers	
			**Name**	**Sequence (5' - 3')**
Ag85A (Rv3804c)	899 bp	pVLExpAg85A4337	Ag85A-5-1	**CGGCAGC**TTTTCCCGGCCGGGCTTGCCGGTG
			Ag85A-N3-1	**CTCCACC**GGCGCCCTGGGGCGCGGGCCCGGT
Ag85B (Rv1886c)	869 bp	pVMExpAg85B4231	Ag85B-5-1	**CGGCAGC**TTCTCCCGGCCGGGGCTGCCGGTC
			Ag85B-N3-1	**CTCCACC**GCCGGCGCCTAACGAACTCTGCAG
MPT63 (Rv1926c)	404 bp	pVMExpMPT634231	MPT63-5-1	**CGGCAGC**GCCTATCCCATCACCGGAAAACTT
			MPT63-N3-1	**CTCCACC**CGGCTCCCAAATCAGCAGATCCTC
MPT64 (Rv1980c)	629 bp	pVMExpMPT644231	MPT64-5-1	**CGGCAGC**GCGCCCAAGACCTACTGCGAGGAG
			MPT64-N3-1	**CTCCACC**GGCCAGCATCGAGTCGATCGCGGA
MTC28 (Rv0040c)	848 bp	pVMExpMTC284337	MTC28-51	**CGGCAGC**GATCCCCTGCTGCCACCGCCGCCTATC
			MTC28-31	**CTCCACC**GCGCGGCGGGACTGGTGTCAGGGT

(B)

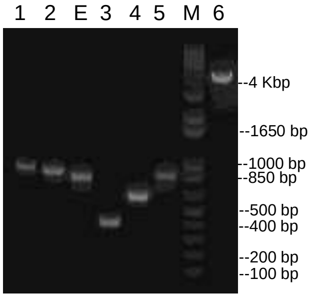

(C)

Construct	Electroporation efficiency (per µg ligated DNA)[a]	Number of clones sequenced	Number of recombinant clones	Cloning efficiency
pVMExpAg85A14367	8×10^6	3	3	100 %
pVMExpAg85B14367	3×10^6	3	3	100 %
pVMExpMPT6314367	5×10^6	3	3	100 %
pVMExpMPT6414367	4×10^6	3	3	100 %
pVMExpMTC2814367	6×10^6	3	3	100 %
