# Supplementary figures and images for: Biotin-tagged proteins: Reagents for efficient ELISA-based serodiagnosis and phage display-based affinity selection
Source: PLoS One. 2018 Jan 23;13(1):e0191315. doi: 10.1371/journal.pone.0191315 (PMC5779676; doi:10.1371/journal.pone.0191315)

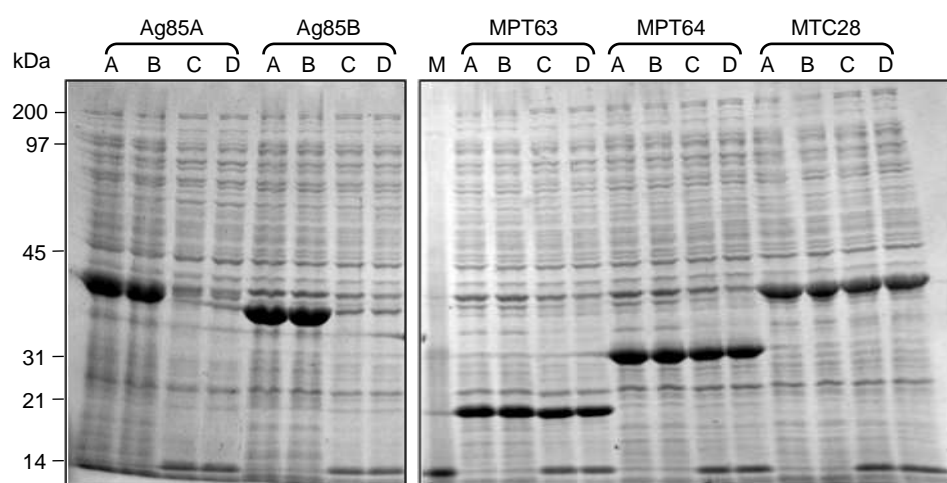

Supplement: S2 Fig — Different sub-cellular fractions were analyzed using 0.1% SDS—8–20% gradient PAGE followed by visualization with coomassie brilliant blue R-250 stain. A, Total cell fraction; B, Total cell fraction after sonication; C, High Speed Supernatant (HSS); D, High-High Speed Supernatant (HHSS). (PDF) [file pone.0191315.s002.pdf]

(A)

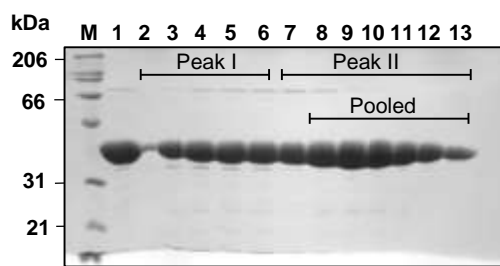

(B)

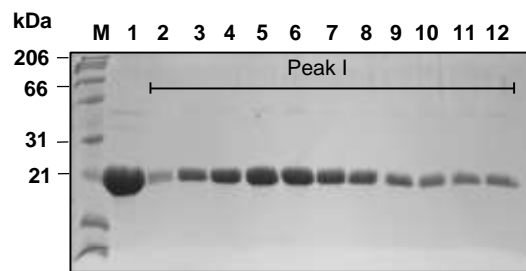

(C)

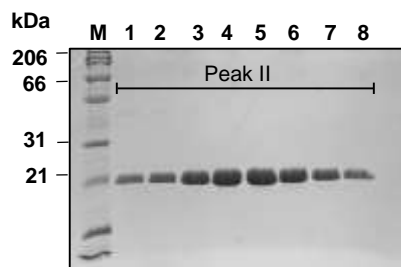

(D)

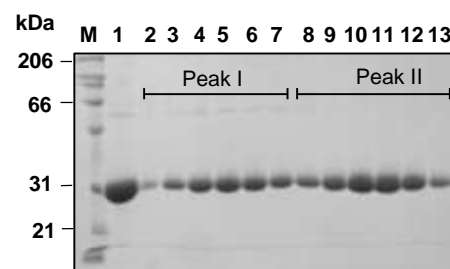

(E)

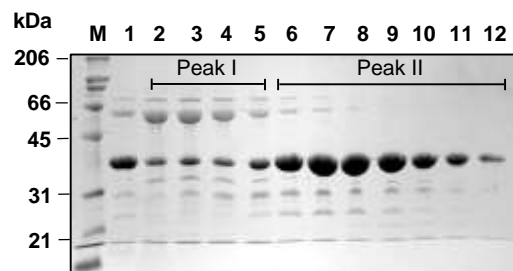

(F)

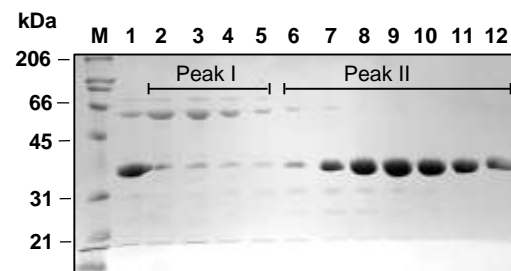

Supplement: S3 Fig — To identify fractions containing desired protein without major contaminants after gel-filtration chromatography, fractions from preparative-scale gel-filtration chromatography (H10-T-MTC28-BAP) or analytical gel-filtration chromatography (other four proteins) were analyzed using SDS-PAGE under reducing conditions, and visualized with Coomassie brilliant blue R-250 staining. For H10-T-MTC28-BAP protein, gel-filtration chromatography of NiFF pool was performed on 480 ml Superdex 75 column (XK 26/100, GE Healthcare). For other proteins, analytical-scale gel-filtration chromatography of 2 ml NiFF pool was performed on 45 ml column (Tricorn 10/600, GE Healthcare) packed with Superdex 75 (H10-T-Ag85A-BAP and H10-T-Ag85B-BAP proteins) or Superdex 200 (H10-T-MPT63-BAP and H10-T-MPT64-BAP proteins), and 1 ml fractions were collected. (A) SDS-PAGE analysis of fractions obtained after gel-filtration chromatography of NiFF pool of H10-T-MTC28-BAP protein. Lane 1, NiFF pool; Lane 2–6, fraction number 4, 6, 8, 10, and 12 constituting peak I; Lane 7–13, fraction number 14, 16, 18, 19, 21, 23, and 24 constituting peak II. (B-F) SDS-PAGE analysis of fractions obtained after analytical gel-filtration chromatography of NiFF pool of other four proteins. (B) H10-T-MPT63-BAP protein (peak I). Lane 1, NiFF pool; Lane 2–12, fraction number 8–18. (C) H10-T-MPT63-BAP protein (peak II). Lane 1–8, fraction number 19–26. (D) H10-T-MPT64-BAP protein. Lane 1, NiFF pool; Lane 2–7, fraction number 7–12 constituting peak I; Lane 8–13, fraction number 19–24 constituting peak II. (E) H10-T-Ag85A-BAP protein. Lane 1, NiFF pool; Lane 2–5, fraction number 4–7 constituting peak I; Lane 6–12, fraction number 8–14 constituting peak II. (F) H10-T-Ag85B-BAP protein. Lane 1, NiFF pool; Lane 2–5, fraction number 4–7 constituting peak I; Lane 6–12, fraction number 8–14 constituting peak II. For all gels, Lane M denotes molecular weight marker in kDa (Broad range, Bio-Rad, Hercules, CA). (PDF) [file pone.0191315.s003.pdf]

(A)

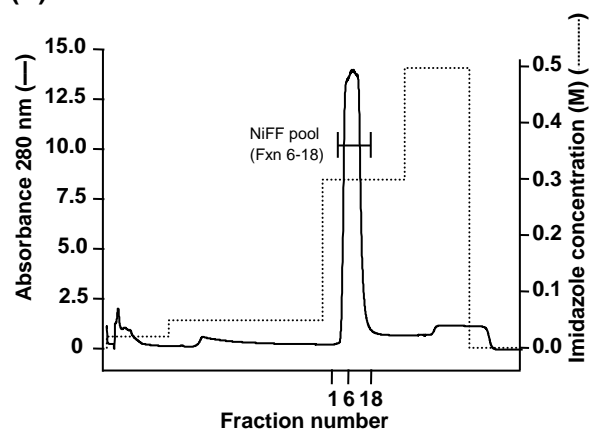

(B)

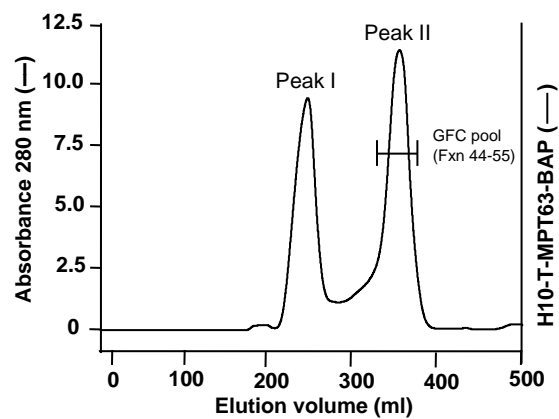

(C)

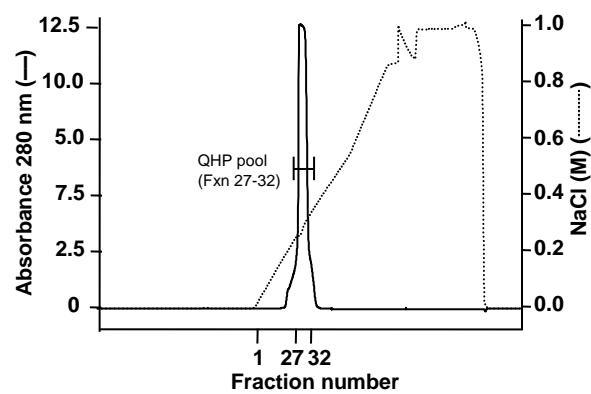

(D)

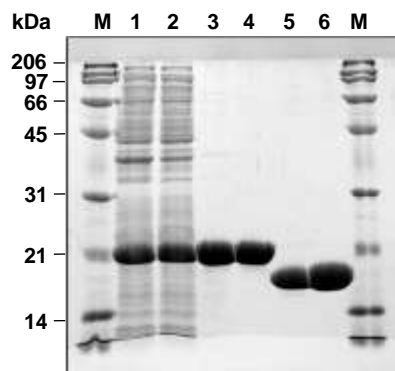

Supplement: S4 Fig — Chromatogram showing (A) Elution profile of H10-T-MPT63-BAP protein on Ni Sepharose Fast Flow (NiFF) affinity column. Fraction numbers 6–18 were pooled (NiFF pool). (B) Elution profile of NiFF pool of H10-T-MPT63-BAP protein on Superdex 200 gel-filtration column. Fraction numbers 44–55 were pooled (GFC pool). The GFC pool was treated with H6-TEV protease to cleave H10 tag from the protein followed by removal of cleaved tag and H6-TEV protease using Ni-affinity chromatography. (C) Elution profile of MPT63-BAP protein on Q Sepharose HP column. Fraction numbers 27–32 were pooled (QHP pool). (D) SDS-PAGE analysis of H10-T-MPT63-BAP protein at different stages during purification. The samples were analyzed by 0.1% SDS-15% PAGE under reducing conditions. The protein bands were visualized with coomassie brilliant blue R-250 staining. Lane M, molecular weight marker, broad range (Bio-Rad, Hercules, CA) (shown in kDa); Lane 1, total cell after homogenization; Lane 2, High-High Speed Supernatant; Lane 3, NiFF pool; Lane 4, GFC pool; Lane 5, NiFF-TT pool (after desalting); Lane 6, QHP pool. (PDF) [file pone.0191315.s004.pdf]

(A)

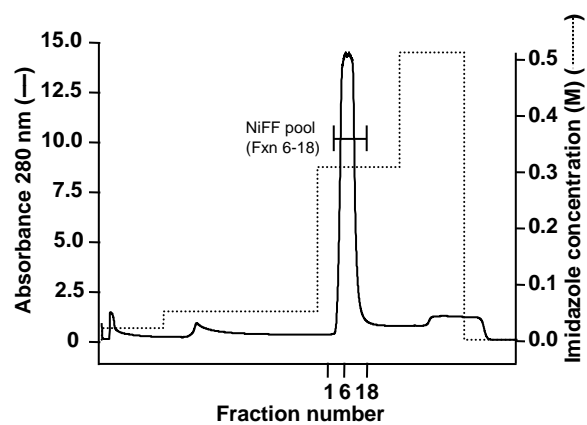

(B)

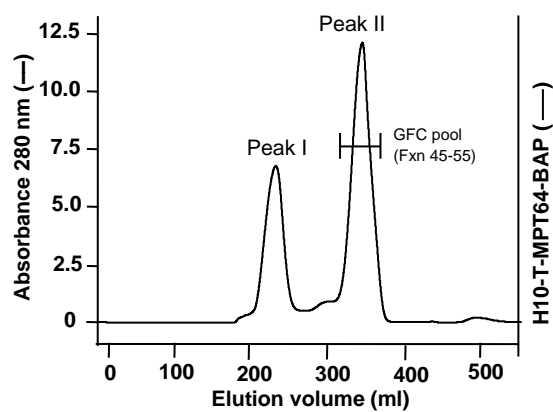

(C)

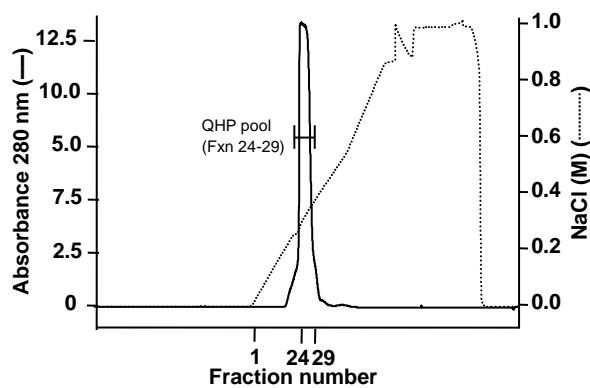

(D)

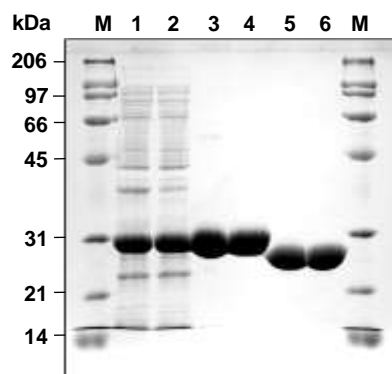

Supplement: S5 Fig — Chromatogram showing (A) Elution profile of H10-T-MPT64-BAP protein on Ni Sepharose Fast Flow (NiFF) affinity column. Fraction numbers 6–18 were pooled (NiFF pool). (B) Elution profile of NiFF pool of H10-T-MPT64-BAP protein on Superdex 200 gel-filtration column. Fraction numbers 45–55 were pooled (GFC pool). The GFC pool was treated with H6-TEV protease to cleave H10 tag from the protein followed by removal of cleaved tag and TEV protease using Ni-affinity chromatography. (C) Elution profile of MPT64-BAP protein on Q Sepharose HP column. Fraction numbers 24–29 were pooled (QHP pool). (D) SDS-PAGE analysis of H10-T-MPT64-BAP protein at different stages during purification. The samples were analyzed by 0.1% SDS-12.5% PAGE under reducing conditions. The protein bands were visualized with coomassie brilliant blue R-250 staining. Lane M, molecular weight marker, broad range (Bio-Rad, Hercules, CA) (shown in kDa); Lane 1, total cell after homogenization; Lane 2, High-High Speed Supernatant; Lane 3, NiFF pool; Lane 4, GFC pool; Lane 5, NiFF-TT pool (after desalting); Lane 6, QHP pool. (PDF) [file pone.0191315.s005.pdf]

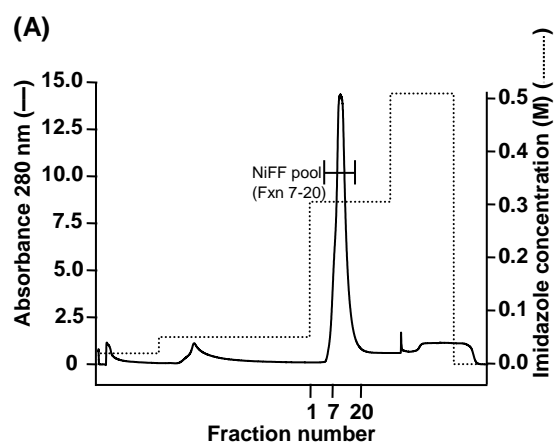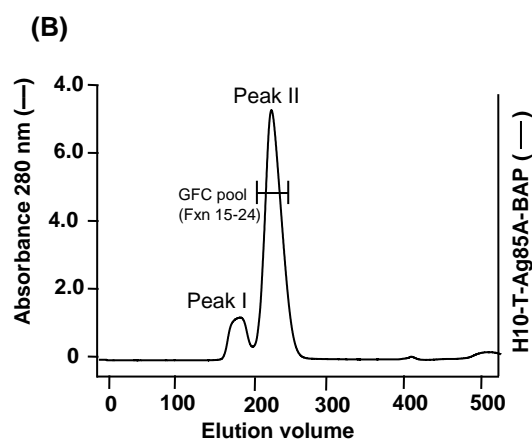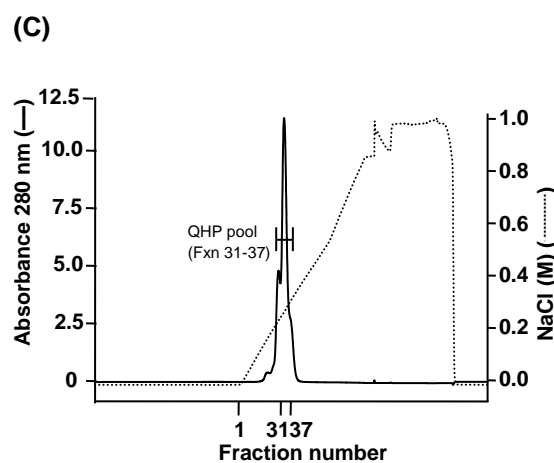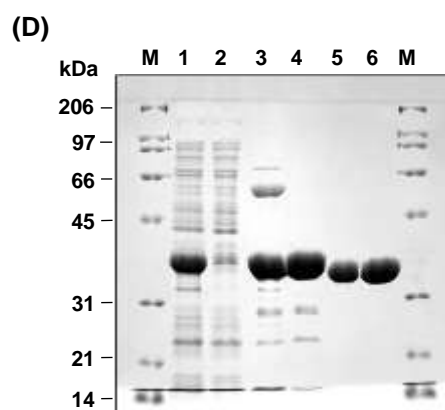

Supplement: S6 Fig — Chromatogram showing (A) Elution profile of H10-T-Ag85A-BAP protein on Ni Sepharose Fast Flow (NiFF) affinity column. Fraction numbers 7–20 were pooled (NiFF pool). (B) Elution profile of NiFF pool of H10-T-Ag85A-BAP protein on Superdex 75 gel-filtration column. Fraction numbers 15–24 were pooled (GFC pool). The GFC pool was treated with H6-TEV protease to cleave H10 tag from the protein followed by removal of cleaved tag and TEV protease using Ni-affinity chromatography. (C) Elution profile of Ag85A-BAP protein on Q Sepharose HP column. Fraction numbers 31–37 were pooled (QHP pool). (D) SDS-PAGE analysis of H10-T-Ag85A-BAP protein at different stages during purification. The samples were analyzed by 0.1% SDS-12.5% PAGE under reducing conditions and visualized with coomassie brilliant blue R-250 staining. Lane M, molecular weight marker, broad range (Bio-Rad, Hercules, CA) (shown in kDa); Lane 1, total cell after homogenization; Lane 2, High-High Speed Supernatant; Lane 3, NiFF pool; Lane 4, GFC pool; Lane 5, NiFF-TT pool (after desalting); Lane 6, QHP pool. (PDF) [file pone.0191315.s006.pdf]

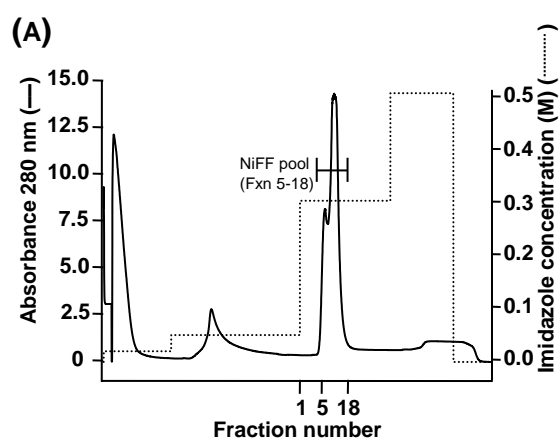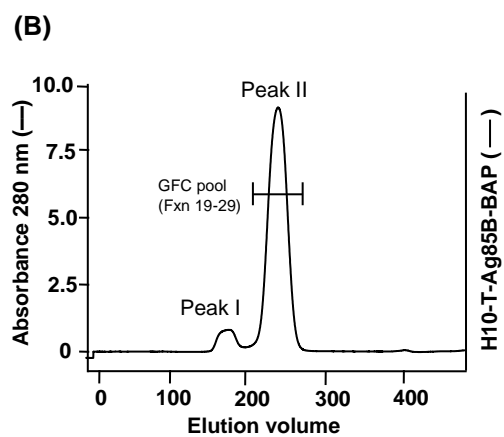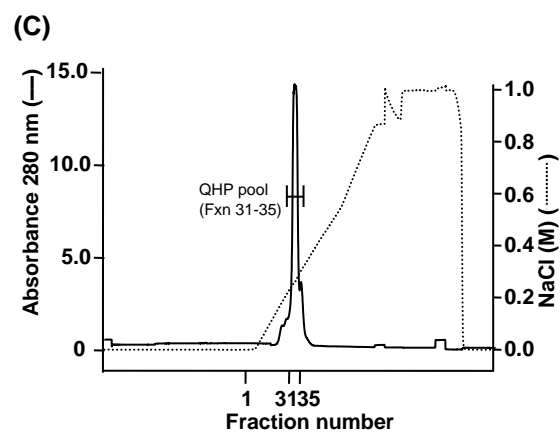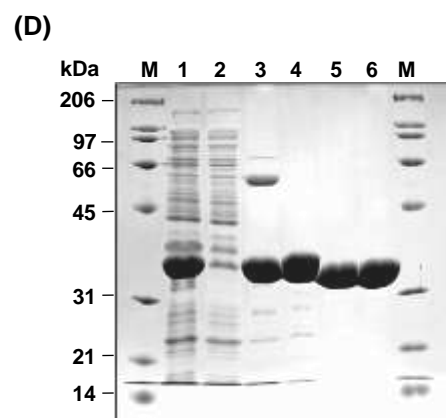

Supplement: S7 Fig — Chromatogram showing (A) Elution profile of H10-T-Ag85B-BAP protein on Ni Sepharose Fast Flow (NiFF) affinity column. Fraction numbers 5–18 were pooled (NiFF pool). (B) Elution profile of NiFF pool of H10-T- Ag85B-BAP protein on Superdex 75 gel-filtration column. Fraction numbers 19–29 were pooled (GFC pool). The GFC pool was treated with H6-TEV protease to cleave H10 tag from the protein followed by removal of cleaved tag and TEV protease using Ni-affinity chromatography. (C) Elution profile of Ag85B-BAP protein on Q Sepharose HP column. Fraction numbers 31–35 were pooled (QHP pool). (D) SDS-PAGE analysis of H10-T-Ag85B-BAP protein at different stages during purification. The samples were analyzed by 0.1% SDS-12.5% PAGE under reducing conditions. The protein bands were visualized with coomassie brilliant blue R-250 staining. Lane M, molecular weight marker, broad range (Bio-Rad, Hercules, CA) (shown in kDa); Lane 1, total cell after homogenization; Lane 2, High-High Speed Supernatant; Lane 3, NiFF pool; Lane 4, GFC pool; Lane 5, NiFF-TT pool (after desalting); Lane 6, QHP pool. (PDF) [file pone.0191315.s007.pdf]

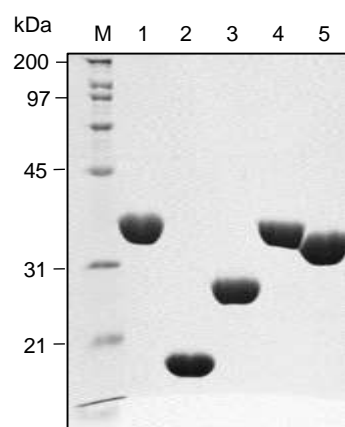

Supplement: S8 Fig — After in vitro biotinylation and removal of H10-BirA enzyme, five microgram of each biotinylated protein was analyzed on 0.1% SDS—12.5% PAGE followed by visualization with Coomassie brilliant blue R-250 staining. Lane M, molecular weight marker, broad range (Bio-Rad, Hercules, CA) (shown in kDa); Lane 1, MTC28-Bio; Lane 2, MPT63-Bio; Lane 3, MPT64-Bio; Lane 4, Ag85A-Bio; Lane 5, Ag85B-Bio. (PDF) [file pone.0191315.s008.pdf]
